# Structural Control of Metabolic Flux

**DOI:** 10.1371/journal.pcbi.1003368

**Published:** 2013-12-19

**Authors:** Max Sajitz-Hermstein, Zoran Nikoloski

**Affiliations:** 1 Systems Biology and Mathematical Modeling Group, Max Planck Institute of Molecular Plant Physiology, Potsdam, Germany; 2 System Regulation Group, Max Planck Institute of Molecular Plant Physiology, Potsdam, Germany; Tel-Aviv University, Israel

## Abstract

Organisms have to continuously adapt to changing environmental conditions or undergo developmental transitions. To meet the accompanying change in metabolic demands, the molecular mechanisms of adaptation involve concerted interactions which ultimately induce a modification of the metabolic state, which is characterized by reaction fluxes and metabolite concentrations. These state transitions are the effect of simultaneously manipulating fluxes through several reactions. While metabolic control analysis has provided a powerful framework for elucidating the principles governing this orchestrated action to understand metabolic control, its applications are restricted by the limited availability of kinetic information. Here, we introduce structural metabolic control as a framework to examine individual reactions' potential to control metabolic functions, such as biomass production, based on structural modeling. The capability to carry out a metabolic function is determined using flux balance analysis (FBA). We examine structural metabolic control on the example of the central carbon metabolism of *Escherichia coli* by the recently introduced framework of functional centrality (FC). This framework is based on the Shapley value from cooperative game theory and FBA, and we demonstrate its superior ability to assign “share of control” to individual reactions with respect to metabolic functions and environmental conditions. A comparative analysis of various scenarios illustrates the usefulness of FC and its relations to other structural approaches pertaining to metabolic control. We propose a Monte Carlo algorithm to estimate FCs for large networks, based on the enumeration of elementary flux modes. We further give detailed biological interpretation of FCs for production of lactate and ATP under various respiratory conditions.

## Author Summary

Insight into the functioning of metabolic control to meet changing demands is a first step in rational engineering of biological systems towards a desired behavior. Metabolic control analysis provides the means to examine the impact of change of reaction fluxes on a specific target flux based on kinetic modeling, but suffers from limitations of the kinetic approach. Here, we introduce and analyze structural metabolic control as a framework to overcome these limitations. We utilize functional centrality, a framework based on the Shapley value from cooperative game theory and flux balance analysis, to determine the contribution of individual reactions to the functions accomplished by a metabolic network. These contributions, in turn, depend on the control exerted on the remaining network. Functional centrality provides the mathematical means to gain further understanding of metabolic control. The potential applications range from facilitating strategies of rational strain design to drug target identification.

## Introduction

Organisms perpetually face changes in environmental conditions. Bacteria may be confronted with variations in oxygen [1] or carbon sources [1], [2], while plants may be exposed to changes in light quality [3] and intensity [4] as well as in availability of carbon [5] and nitrogen [6]. Animals, on the other hand, may have to cope with shifts in temperature [7]. To ensure survival, growth, and reproduction, organisms adapt to these perturbations. The adaptation is likely to be reflected in physiological changes across some or all levels of biological organization, from single cells to tissues, organs, and the organism itself. The molecular mechanisms of adaptation involve concerted action through gene regulatory and signaling interactions which ultimately induce the modification of the metabolic state to meet the change in metabolic demands [8]–[10]. Such transitions in metabolic state are not only the response to shifts in environmental conditions, but also occur upon changing demands during development, *e.g.*, in the switch from sink to source leaf [11] or during the cell cycle [12], as well as during metabolic cycles [13].

The metabolic state is determined by the concentration of metabolites and fluxes of biochemical reactions interconverting the metabolites. Changes in metabolite concentrations are governed by alterations of reactions' fluxes which depend on the concentration of reactants themselves. Moreover, fluxes are often under tight condition-specific regulation by means of varying enzyme concentrations/activities via transcriptional and (post)translational modifications [14], [15] as well as through metabolic and allosteric regulation via various effectors (*i.e.*, activators and inhibitors) [16]. However, reaction fluxes are not equally regulated. For instance, regulation targeted at specific enzymes has been observed in facultative anaerobic bacteria upon change from oxic to anoxic environment [17]. The adaptation processes resulting in a preferable metabolic state are crucial to guarantee functioning and, hence, survival. Therefore, the ability of metabolism to adapt to changing conditions, via regulation of its state, can be regarded as a potential for controlling this complex dynamical system.

A dynamical system is called controllable if it can be driven from an initial state to a desirable state by the manipulation of a suitable set of variables [18]. Since regulation of the transition between metabolic states is targeted at enzymes and corresponding reactions, reaction fluxes can be considered as the metabolic control variables. Biochemical reactions do not act in isolation and, hence, the process of metabolic adaptation is a result of complex interactions among the system components (*i.e.*, metabolites and enzymes). Therefore, assessing the extent to which reaction fluxes have to be manipulated to control the metabolic state is a nontrivial task, as metabolic control is a systemic phenomenon [19].

A recent study has shown that, in general, multiple metabolites have to be manipulated to control metabolic networks [20]. While it remains unclear to what extent this holds if control is exerted on reaction fluxes obeying stoichiometric and physiochemical constraints, it indicates that metabolic control is the result of cooperative action. Furthermore, metabolic control can be assumed to depend on the preferred metabolic state an organism aims to attain, which may exhibit altered concentrations [21] or modified fluxes [22].

Mathematical models, capturing the interactions of the most relevant components, provide tractable means for understanding metabolic control [23], [24]. A frequently utilized framework is metabolic control analysis (MCA) [25]–[27] which enables the examination on the basis of kinetic modeling. It has generated diverse insights in metabolic control and served as the basis of rational strain design in metabolic engineering [28]–[31]. However, the limited availability and accuracy of condition-specific kinetic parameters render the applicability of kinetic models to metabolic processes of small scale [32].

On the other hand, structural modeling, although neglecting the details of the kinetics, has proven to be successful in describing and predicting phenotypes across different organisms, from bacteria to plant and human [33]–[40]. Nevertheless, a computational framework for quantifying and investigating metabolic control based on structural models is currently lacking.

Here, we introduce *structural metabolic control* as a framework to examine the effect of the manipulation of a metabolic network (via enabling and disabling the utilization of reactions) on the operation of selected metabolic functions.

In structural modeling, metabolic functions are equivalent to combinations of fluxes and can correspond to the objective function of flux balance analysis (FBA). The synthesizing capacity of a metabolic function can then be determined by optimizing the objective upon the given constraints [33], [41]. We examine structural metabolic control by employing functional centrality (FC) [42] which quantifies a reaction's contribution to the synthesizing capacity of a metabolic function via a modified version of the Shapley value from cooperative game theory [43], [44]. This framework integrates the potential interactions between reactions by considering the multiplicity of subnetworks capable of performing the metabolic function. We demonstrate that FC is suitable for elucidating metabolic control and for identifying reactions as potential sites of control. Moreover, this framework allows investigations of the dependency of metabolic control on the environmental conditions, providing insights in the environment-specificity of the distribution of control among reactions. From a computational point of view, we propose an approximation algorithm based on Monte Carlo sampling which extends the applicability of FC to metabolic networks of large size. The algorithm is based on the calculation of elementary flux modes (EFMs), inheriting its computational complexity.

In addition, we provide a comparative analysis of FCs and other structural approaches related to metabolic control, namely, control-effective fluxes (CEFs) [45], reaction participation in EFMs [46] and flux couplings [47]. To this end, we use a model of central carbon metabolism of *Escherichia coli* (*E. coli*) [22] in combination with a Monte Carlo multiple knockout study. Furthermore, we examine structural metabolic control with respect to lactate and ATP production in *E. coli* for different respiratory states by FC, and discuss and biologically interpret the variation due to metabolic functions and environmental conditions. Our results indicate that FC can further expand the current understanding of metabolic control.

## Results

We first discuss the available approaches to examine control of metabolic flux in the established modeling frameworks. Metabolic control analysis, in particular flux control coefficients, is considered and the framework of structural metabolic control is introduced. We briefly describe structural approaches related to metabolic control, *i.e.*, flux couplings, reaction participation in EFMs, CEFs, and provide a more detailed description of FC. The latter is shown to be superior to examine structural metabolic control compared to the other structural approaches in an FBA-based multiple knockout study. We then analyze the dependency of FC on metabolic function as well as on environmental conditions. Finally, we compare predictions of transcript level alterations by FCs and CEFs, and analyze the association of the two measures with the number of transcription factors acting on individual enzyme catalyzed reactions.

### Examination of metabolic control

Mathematical modeling of metabolic processes can be divided essentially into two distinct conceptual approaches: *kinetic modeling* and *structural modeling* [48]–[50].

Kinetic modeling provides the means to describe and predict both steady-state and transient behavior of metabolite concentrations and reaction fluxes prevalently via ordinary differential equations. However, kinetic models are based on largely unavailable or unreliable information on kinetic rate laws, on the nature of regulation processes and on values for the kinetic parameters [32], [51]. Already the modeling of small and well-investigated pathways, such as the Calvin-Benson cycle, has shown to be challenging [51].

Structural modeling circumvents the problem of uncertainties with regard to kinetic information by relying only on structural information. It utilizes reaction stoichiometry, reaction directionalities obtained from basic thermodynamics, and flux boundaries [49], [50], [52], which could often be confined through integration of condition-specific high-throughput data [53]. The resulting metabolic network then is prevalently examined either by determining *particular* steady-state flux distributions guided by optimization principles [33], [41] or by characterizing *all* steady-state fluxes via the set of all minimal nondecomposable functional (*i.e.*, flux carrying) pathways [45], [46]. It is apparent that the price for this simplification is the restriction to steady-state behavior and the potential occurrence of physiologically unrealistic flux distributions.

#### Metabolic control analysis

Metabolic control analysis (MCA) is a mathematical framework which facilitates investigations of metabolic control in kinetic models by a sensitivity analysis of a reference steady state [25]–[27]. It defines *flux control coefficients* (FCCs) which quantify the effect of change in an enzyme's activity on metabolic flux and which have been proposed as a measure of a reaction's potential to control the flux [27].

FCCs possess various properties facilitating the examination of metabolic control. Most important, FCCs capture system-wide interactions as they are determined by evaluating the systemic response on local perturbations. By the choice of a reference state, environmental conditions and metabolic demands are incorporated, such that, in principal, FCCs enable a systematic analysis of the specificity of metabolic control. The main result of MCA tying FCCs to metabolic control is the summation theorem which states that the whole of FCCs pertaining to a specific metabolic flux sum up to one [27], [50]. Thus, FCCs have been suggested to quantify “share of control” [25], [27].

The application of MCA to kinetic models has generated valuable insights and predictions for the control of metabolic flux [28], [29]. Moreover, FCCs have successfully been utilized for targeted metabolic engineering strategies to improve production of certain biochemicals, *e.g.*, increasing diacethyl production in *Lactococcus lactis* [30] or ethanol production from glycerol in *E. coli* [31]. Nevertheless, despite the appealing properties of FCCs, successful applications to rational strain design are scarce.

Some critical problems exist in the utilization of MCA. The choice of a reference state in accordance with experimental results is a prerequisite for its application. Therefore, and due to the inherently nonlinear nature of metabolism (and of the abstracted kinetic models), the results are only valid for small perturbations around the reference state which hampers elucidating general system properties. Furthermore, while it is possible to built large-scale kinetic models and even kinetic models of genome scale [54], [55], these models predominantly rely on the estimation of a large set of parameters and apply usually generalized kinetics such as linlog kinetics [54]–[56]. The dynamics of these models then normally pertain only very closely to the chosen reference state [54] which undoubtedly results in a bias of MCA outcomes. Besides, FCCs can attain negative values and, due to the summation theorem, other reactions can then be assigned FCCs larger than one [57]. This renders the interpretation of FCCs as a share of control difficult.

#### Structural metabolic control

Examination of metabolic control based on structural modeling constitutes a possibility to transcend the inherent problems of kinetic modeling. Up to now, there exists no framework for systematic analysis of metabolic control based on structural modeling, but there are several approaches which examine structural properties that can be related to metabolic control.

Identifying sites of control in a given metabolic network requires quantification of the contribution of the network components to the functionality of the network as a whole. Here, the sites of control are reactions, since structural approaches deal with and can quantify the distribution of reaction fluxes in the network. The notion of control varies with the considered network functionality. Thus, examination of structural metabolic control requires specification of *metabolic functions* representing fluxes, ranging from flux through single reactions, like the synthesis of a specific target metabolite, to composite fluxes, *e.g.*, the sum of fluxes through ATP producing reactions. The degree of a specific metabolic functionality of a network is given by the corresponding synthesizing capacity. Here, the metabolic function corresponds to the objective function of FBA (see Methods section) and we determine the synthesizing capacity by maximizing this function.

In the framework of structural metabolic control, the control of a metabolic function is exerted by activation and deactivation of reactions. Each reaction is then described by its *status*, *i.e.*, active or inactive. While an active reaction may carry nonzero flux, this is not the case for an inactive reaction. By deactivation of a reaction its status switches from active to inactive, which is equivalent to its removal from the network. Activation of a reaction denotes the reverse process. Note that activation of a reaction does not imply that its flux is forced to be nonzero.

A subnetwork of the investigated network is then an assignment of particular status (*i.e.*, active/inactive) to each reaction in the metabolic network. Given a metabolic function, a subnetwork is called *functional* if it is capable to carry nonzero flux with respect to the metabolic function and the environmental conditions.

The aim of this study is to identify the reactions with the largest influence on a metabolic function. These reactions are *potential* sites of control, since the effect of the manipulation depends on the distributed action on several reactions. The potential effect of a reaction on metabolic function is referred to as its *structural metabolic control capability*.

#### Approaches related to structural metabolic control

Several structural modeling approaches exist which describe properties of reactions that are linked to structural metabolic control.

Flux coupling analysis derives dependencies between pairs of fluxes due to steady-state constraints employed in FBA, a detailed description is given in [47]. Two reactions are said to be coupled, if a nonzero flux through one reaction implies a nonzero flux through the other reaction. Coupled reactions have been shown to exhibit significantly higher correlations of their *in vivo* fluxes in comparison to uncoupled reactions [58]. We examine the extent to which the *coupling degree*, the number of fluxes coupled to the flux of a specific reaction, is related to structural metabolic control capabilities.

EFM analysis allows a unique decomposition of a metabolic network into minimal nondecomposable pathways capable of carrying nonzero flux at steady state [38], [46], [59]. They can be considered as a *functional decomposition* which allows identification of relations between the reactions and the metabolic functions. EFMs indicate which reactions may cooperatively interact to accomplish a metabolic function of the network. Deactivation of a reaction results in the deactivation of all EFMs utilizing that reaction [59]. Reaction participation in extreme pathways, which are closely related to EFMs, has already been proposed as a potential indicator of metabolic regulation [60]. We examine the frequency of reaction participation in EFMs restricted to a metabolic function and specific environmental conditions. Furthermore, we consider weighting EFMs by the production yield to increase the contribution of more productive routes.

CEFs utilize EFMs to quantify the importance of individual reactions for efficient and flexible operation of metabolic functions and have been introduced to elucidate cellular regulation [45]. The calculation of CEFs is described in the Methods section. Correlations have been found between ratios of transcript levels and ratios of CEFs upon substrate change, indicating their utility for the identification of regulatory patterns [45], [61], [62].

FC is a framework to quantify the relevance of a reaction to the synthesizing capacity of a metabolic function which is determined by FBA [42]. To this end, it integrates the change in optimal production upon activation/deactivation of the reaction for all functional subnetworks, thus capturing all possible interactions with the remaining system. Integration of the individual contributions by FC is required to fulfill axiomatic properties to guarantee a *fair* assignment of the reaction's relevance for optimal operation: *efficiency*, *marginalism* and *internal symmetry* (see Supplementary Text S1). These requirements result in a mapping of the contributions on a scalar value which is equivalent to a modified version of the Shapley value [44] from cooperative game theory. The Shapley value has been applied in its original version to determine the contribution of individual entities in diverse fields of science [63]–[65]. The calculation of FCs is described in the Methods section. The framework has been successfully applied to quantify the relevance of reactions for the metabolic function of ATP production in a model of monosaccharide metabolism of *E. coli* [42].

FC represents a division of the synthesizing capacity of a metabolic function among the considered reactions with respect to their contribution. Therefore, it can be interpreted as an assignment of “share of control” in analogy to MCA's summation theorem [27]. High FC of a reaction then suggests large structural metabolic control capability.

The application of FCs has been limited to small networks due to the combinatorial complexity arising from the number of functional subnetworks. Here, we present an estimation algorithm that enables computation of FCs for large networks based on Monte Carlo sampling (see Methods section). We utilize resampling to determine the errors.

The estimation of FCs exploits the fact that the set of functional subnetworks can be derived from the set of EFMs (see Methods section). Hence, it initially requires the enumeration of the EFMs which are capable of the examined metabolic function and in accordance with environmental conditions. This usually implies computation of all EFMs. The calculation of EFMs in genome-scale networks is hampered due to combinatorial explosion and, therefore, represents a bottleneck to the estimation of FCs as it is presented in this study (which is also the case for the other EFM based measures). To our knowledge, the largest set of EFMs has been calculated for a compartmentalized model of central metabolism of *Saccharomyces cerevisiae*. That model comprises central carbon metabolism and amino acid synthesis pathways, which altogether encompass 230 reactions [66]. In the discussion, we briefly describe potential extensions and modifications to push the limit of the approach up to genome scale but which are out of the scope of this study.

### Analysis of structural metabolic control

**Figure 1:**
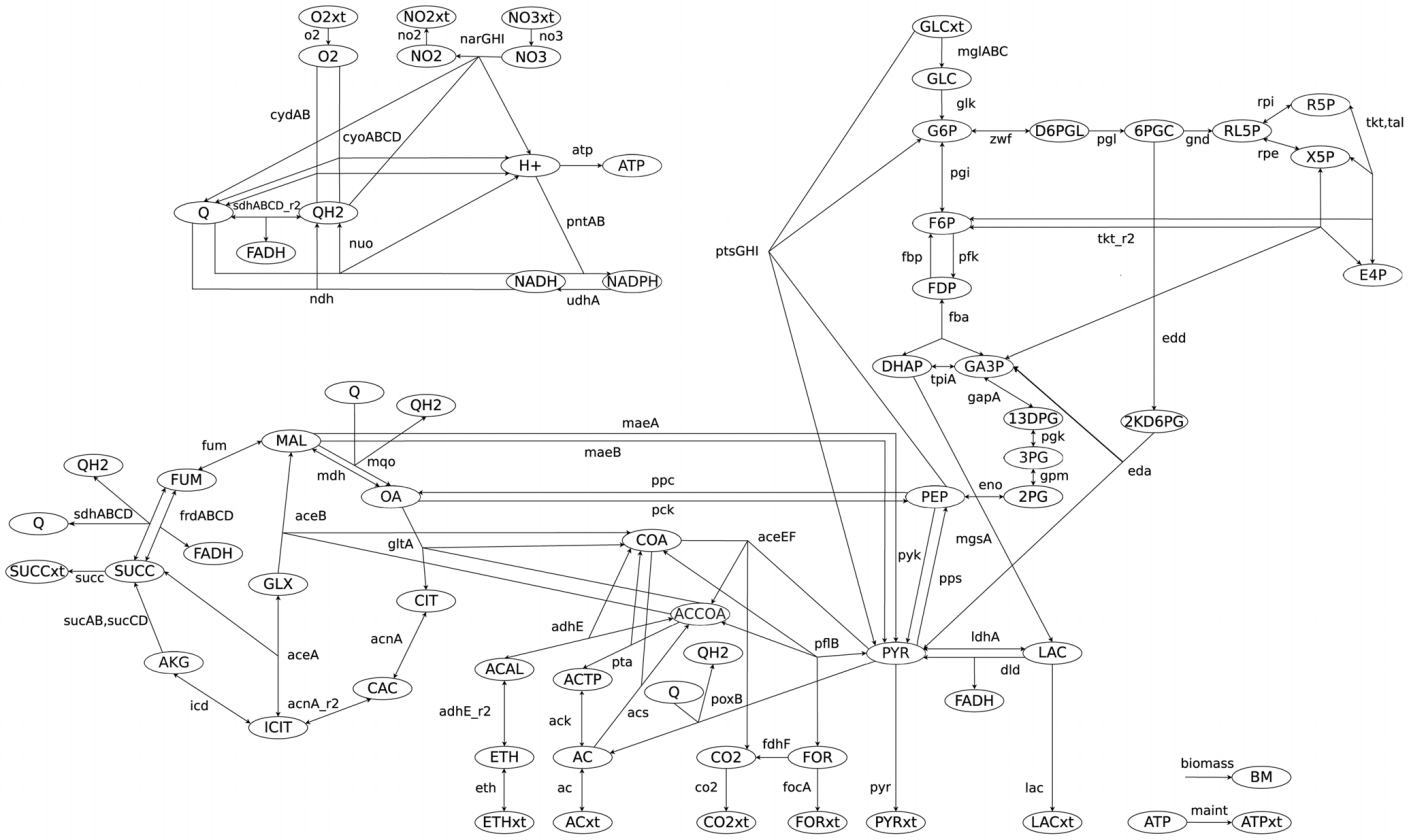
Metabolic network of central carbon metabolism of *E. coli*. Reactions and metabolites are listed in Supplementary Tables S11 and S12.

We examine structural metabolic control on the example of central carbon metabolism of *E. coli*. For this purpose, we utilize a stoichiometric model introduced by Schütz *et al.* [22] (see Methods section and Figure 1). We analyze the potential of the presented structural measures, *i.e.*, coupling degree, reaction participation in EFMs, CEFs and FC, to identify potential sites of control. To this end, we conduct a comparative *in silico* knockout study for all described measures, demonstrating the superior capability of FC to identify potential sites of control.

Structural metabolic control ought to depend on metabolic function. We determine FCs for two metabolic functions, lactate and ATP production, under aerobic conditions and give a detailed biological interpretation of the results. Environmental conditions should also be reflected in the structural metabolic control capabilities. We examine the changes in FCs upon change in environmental conditions for lactate and ATP production and give detailed biological interpretation. Furthermore, we utilize CEFs and FCs to predict changes of transcript levels upon switch of substrate, and analyze the association of the two measures with number of transcription factors.

#### Multiple knockout study

We conduct an *in silico* Monte Carlo multiple knockout study to quantify the capability of the various measures to examine structural metabolic control. Reaction activation/deactivation has already been proposed as the means of controlling metabolic flux in the framework of structural metabolic control. The analysis of random reaction deactivation offers an unbiased way to statistically infer the effect of controlling reactions. Reaction activation, on the other hand, would require uniform sampling of random functional subnetworks which is difficult to achieve. Multiple knockouts enable quantifying the synergetic effect of controlling several reactions simultaneously. The reactions to be knocked out are chosen with probabilities proportional to the values assigned by the various measures.

We examine production of biomass as metabolic function under conditions of aerobic respiration, nitrate respiration and fermentation (see Methods section). The biomass reaction is a composite drain comprising all biomass constituting metabolites or biomass precursors in experimentally determined proportions [67]. It can be linked to growth rate in unicellular organisms and has been shown to yield good predictions of prokaryotic phenotypes [22], [35]–[37], [67]. It is a natural objective of central carbon metabolism and incorporates a wide range of the model's complex functionality.

Knockouts are sampled without replacement from the probability distributions
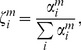
(1)whereby 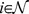 denotes a reaction from the set of considered reactions 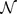 and 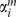 its value assigned by the measure 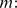 functional centrality (FC), number of reaction participations in biomass producing EFMs (unweighted: EFM, and weighted by biomass yield: 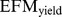), coupling degree with respect to flux coupling analysis (FCA) and control-effective fluxes (CEF); further, we utilize 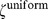, denoting a probability distribution assigning the knockout of every considered reaction the same probability (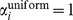). The values obtained by the respective measures are to be found in Tables S1, S2 and S3.

Knockouts are realized 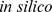 by constraining the respective reactions to zero flux in the FBA. This is equivalent to FBA on the subnetwork resulting from removing the knockout reactions from the network. We record biomass production for up to 25 simultaneous knockouts with a sample size of 200,000. A knockout resulting in no FBA solution corresponds to a nonfunctional subnetwork. In that case, we assign zero biomass production.

**Figure 2:**
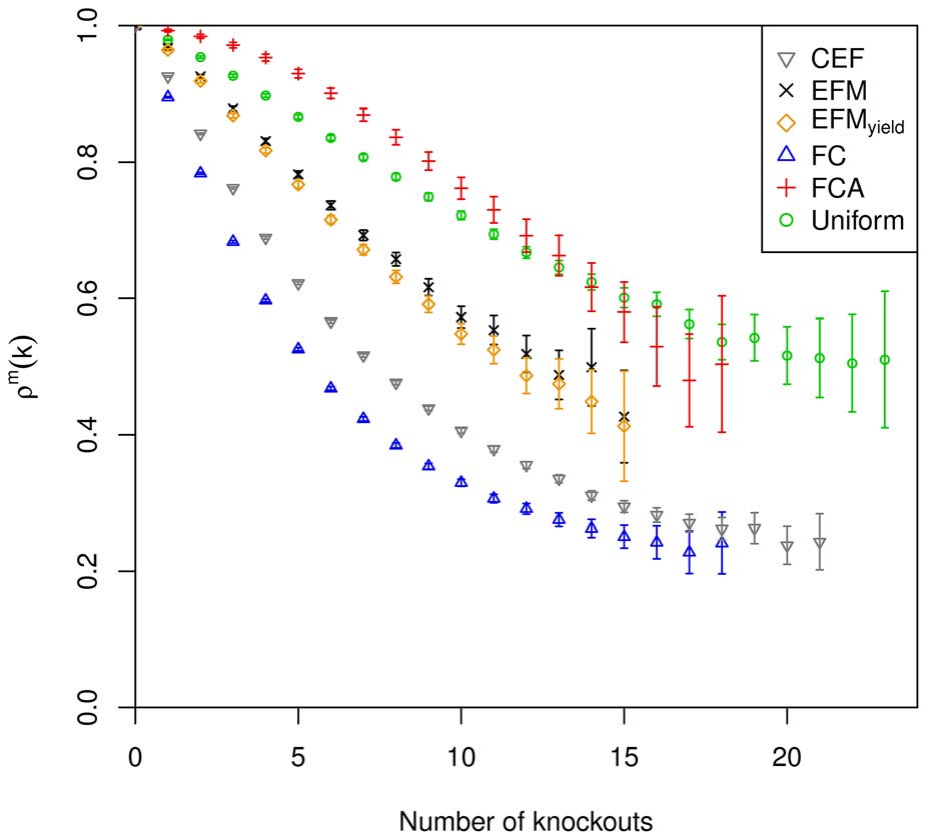
KO-reduced functionality of biomass production under conditions of aerobic respiration. Errorbars are provided for all 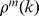 and indicate 95% confidence intervals. Results are only depicted for a relative standard error smaller than 10%.

**Figure 3:**
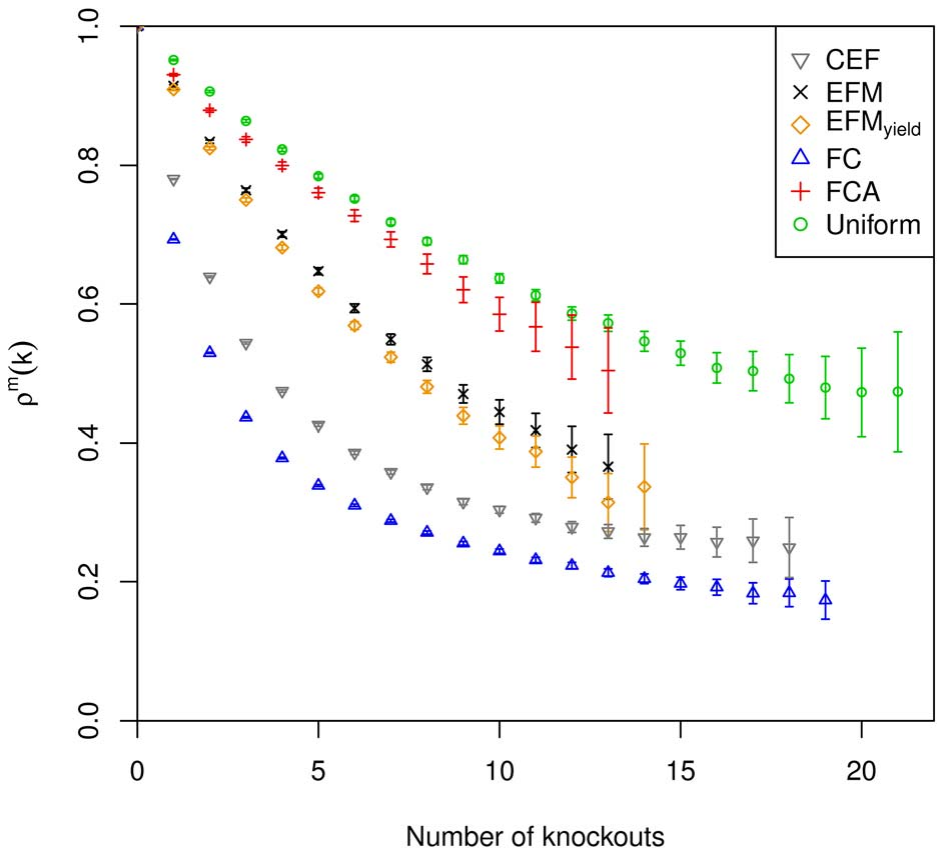
KO-reduced functionality of biomass production under conditions of nitrate respiration. Errorbars are provided for all 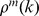 and indicate 95% confidence intervals. Results are only depicted for a relative standard error smaller than 10%.

**Figure 4:**
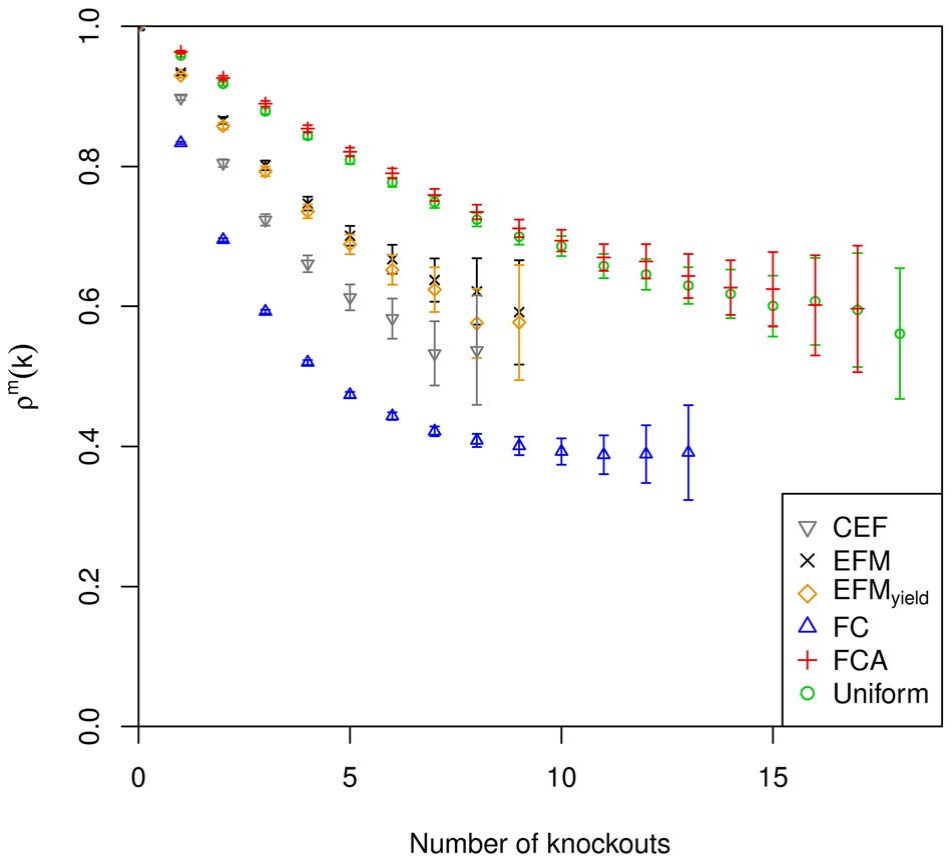
KO-reduced functionality of biomass production under conditions of fermentation. Errorbars are provided for all 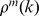 and indicate 95% confidence intervals. Results are only depicted for a relative standard error smaller than 10%.

We define a new statistical measure to quantify the effect of multiple reaction deactivations on a metabolic function, here biomass production. In particular, we examine the relative synthesizing capacity of the metabolic function, meaning the synthesizing capacity after 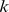 knockouts divided by the synthesizing capacity for zero knockouts. The *KO-reduced functionality* 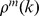 denotes the average of the relative synthesizing capacity of a metabolic function per functional subnetwork after 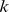 knockouts for measure 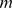,
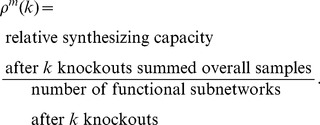
(2)If knockouts of genes are chosen with probabilities 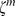 according to the measure 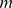 of structural metabolic control capabilities, 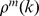 captures the reduction of the synthesizing capacity of a metabolic function in a functional system with respect to the considered measure. The KO-reduced functionality 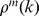 is an estimate of the expectation value which would be obtained for infinite sampling. Therefore, the accuracy of 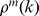 depends on the utilized sample size. We provide a detailed statistical derivation of confidence intervals of 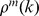 in Supplementary Text S2. The deviation of calculated values is considered significant if the 95% confidence intervals do not overlap. The confidence intervals are shown in Figures 2, 3 and 4 in terms of error bars. The uniform sampling serves as the reference: KO-reduced functionality indicates that the examined measure captures structural metabolic control capabilities with respect to the examined metabolic function, if the decrease is significantly larger than observed for uniform sampling. The extent of the deviation serves as a benchmark of the capability to identify potential sites of control.

In the following we describe the effects of utilizing the various measures on the KO-reduced functionality and, therefore, their capability to capture structural metabolic control.

The KO-reduced functionality according to the coupling degree shows larger values compared to uniform sampling for up to 13 knockouts under aerobic conditions (Figure 2). For higher numbers of knockouts the values are smaller than the values for uniform sampling; however, as the confidence intervals overlap, the differences are not significant. Under conditions of nitrate respiration, KO-reduced functionality is constantly smaller than for uniform sampling (significant deviation up to 11 knockouts), but the difference is minor compared to the other measures (Figure 3). In the fermentative environment, no major significant differences to uniform sampling can be observed, the curves are overlaid (Figure 4). Together this indicates low potential of coupling degree to capture structural metabolic control capabilities. (Flux couplings are determined with the requirement for nonzero biomass formation larger than 1% of the optimal value.)

Knockouts with respect to participation in biomass producing EFMs result in a KO-reduced functionality significantly below that of uniform sampling for 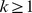 in all settings (Figures 2, 3 and 4). The results are comparable for the three environmental conditions. Additional weighting by biomass yield results in smaller values of KO-reduced functionality compared to the unweighted case. Both variants of reaction participation indicate some potential to capture structural metabolic control capabilities.

KO-reduced functionality for knockouts with respect to CEFs shows significantly smaller values compared to the results of the reaction participation measures under conditions of aerobic and nitrate respiration (Figures 2 and 3). Under fermentative conditions the difference to reaction participation is less pronounced (Figure 4). Nevertheless, CEFs show considerable potential to capture structural metabolic control capabilities.

Knockouts with respect to FC yield almost significantly smaller values of KO-reduced functionality compared to all other measures under all considered environmental conditions (Figures 2, 3 and 4). The only exception is found for aerobic conditions where the difference to CEFs is not significant for more than 16 knockouts. The stability of outcomes together with the large decline in synthesizing capacity indicates its potential to capture the structural metabolic control capabilities of individual reactions.

Comparing the impact of the various measures on KO-reduced functionality indicates FC to be the preferential measure to examine structural metabolic control.

#### Dependence on metabolic function

The reactions' structural metabolic control capabilities should depend on the metabolic function. We calculate FCs with respect to two different metabolic functions to examine the relationship. We analyze lactate production and ATP production under aerobic conditions and compare both to each other and to biomass production. In the examination of metabolic functions other than biomass production, we disable the biomass reaction since it constitutes an artificial drain.

Lactate is a major fermentation product in *E. coli* [68] and its production is also of biotechnological relevance [69]. The synthesis of lactate is relatively simple compared to other metabolic functions such as biomass production.

We observe that the reactions with highest FCs form a minimal pathway with glycolysis at its core (Figure S1; highest ranked reactions are given in Table 1, all ranks are shown in Table S4 and the complete values are given in Table S5). All participating reactions obtain similar FCs. Initially, glucose is incorporated and converted to glucose 6-phosphate by the phosphotransferase system *ptsGHI*. The glucose 6-phosphate is then converted to triose phosphates, dihydroxyacetone phosphate and glyceraldehyde 3-phosphate, by the glycolytic reactions *pgi*, *pfk* and *fba*. The glyceraldehyde 3-phosphate follows glycolysis down via *gapA*, *pgk*, *gpmA* and *eno* to phosphoenolpyruvate which is further transformed to pyruvate by *ptsGHI*. The pyruvate then is converted to lactate by lactate dehydrogenase *ldhA*. The dihydroxyacetone phosphate is directly converted to lactate via *mgsA* (in the utilized model [22] the reaction *mgsA* denotes a lumped reaction converting dihydroxyacetone phosphate directly to lactate with methylglyoxal as intermediate). The utilization of *ptsGHI* enables a shorter pathway accounting for the same overall reaction as the combination of *mglABC*, *glk* and *pyk*. It is apparent that manipulation of reactions of this minimal pathway will largely affect lactate production. It should be noted that *lac* is the only essential reaction for lactate production in this model with glucose as a substrate.

**Table 1:** Reactions with highest ranking according to functional centrality.

LAC (O_2_)		ATP (O_2_)		ATP (NO_3_)		ATP (Ferm)	
Rank	Reaction ID	Rank	Reaction ID	Rank	Reaction ID	Rank	Reaction ID
1	*eno*	1	*atp*	1	*no*2	1	*fba*
	*gapA*	2	*co*2		*narGHI*	2	*pfk*
	*gpm*	3	*o*2		*no*3		*tpiA*
	*lac*	4	*nuo*	2	*atp*	3	*pgi*
	*ldhA*	5	*cyoABCD*	3	*co*2	4	*pyk*
	*pgk*	6	*gltA*	4	*nuo*	5	*eth*
	*ptsGHI*		*acnA*	5	*acnA_r*2		*adhE*
2	*fba*		*acnA_r*2		*acnA*		*adhE_r*2
	*pfk*	7	*fum*	6	*gltA*	6	*ac*
	*pgi*	8	*ac*	7	*fum*		*ack*
							*pta*

Rankings are shown for the metabolic functions of lactate production (LAC) and ATP production (ATP) under conditions of aerobic respiration (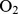), nitrate respiration (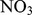) and fermentation (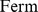). Reactions with highest FCs obtain lowest numbering.

The observed FCs are similar for other respiratory conditions (see below) and in line with the experimental results of Zhou *et al.* [70] and Tian *et al.* [71], associating reactions showing low FC with reactions whose knockouts do not affect or even increase lactate production. Zhou *et al.* could show that the deletion of genes corresponding to reactions accounting for production of competing by-products (*i.e.*, *ack*, *pta*, *pps*, *pflB*, *dld*, *poxB*, *adhE*, *frdA*) leads to an increase in lactate production [70]. All of these reactions obtain extremely low FCs. For the same *E. coli* strain and experimental conditions, Tian *et al.* have shown that overexpression of *ldhA*, which is among the reactions obtaining highest FCs, further increases lactate production [71].

ATP is the prevalent energy equivalent in metabolism and its production, meaning the formation from ADP and Pi, is a vital metabolic function. The energy which is set free in specific reactions is used to produce ATP, as it is the case in glycolysis. On the other hand, the such stored energy can be utilized to drive energy demanding processes like amino acid synthesis and polymerization [72]–[74]. Aerobic respiration represents in *E. coli* the most efficient way to produce ATP from carbohydrates which are fully oxidized to carbon dioxide [8]. It has been shown in *E. coli*, that ATP production is linearly correlated with growth rate under certain conditions [75], [76]. Maximization of ATP production has also successfully been applied to predict physiological states via FBA [22], [34].

We observe that the ATP synthase reaction *atp* obtains by far the highest FC followed by carbon dioxide export *co2*, oxygen import *o2*, NADH dehydrogenase I catalyzed reaction *nuo* and cytochrome *bo3* oxidase catalyzed reaction *cyoABCD* (Figure S2; highest ranked reactions are given in Table 1, all ranks are shown in Table S4 and the complete values are given in Table S6). Despite carbon export, all of these are involved in the electron transport chain (ETC), which is central to efficient ATP production in aerobic respiration. In the ETC, electrons are transferred from the reducing equivalents NADH, which have been generated during carbohydrate oxidation, *e.g.*, by pyruvate dehydrogenase *aceEF*, to a terminal electron acceptor. The free energy of this process is used to pump protons from cytoplasm to periplasm creating a proton gradient. Here, ATP synthase *atp* is the central reaction which uses the resulting proton-motive force to produce ATP from ADP [77].

The reaction *co2* obtains the second highest FC. It exports carbon dioxide which is the end product of carbohydrate oxidation. Carbohydrates cannot be fully oxidized if the resulting carbon dioxide is not excreted. In that case, other end products which still contain extractable chemical energy like ethanol would have to be released.

In the ranking, the carbon dioxide export is followed by the oxygen import reaction *o2*. The reaction provides the oxygen which is utilized as terminal electron acceptor in the ETC enabling full oxidization of carbon hydrates to carbon dioxide.

It should be noted that oxygen and carbon dioxide diffuse across the periplasmic membrane. Therefore, while the transport of these metabolites is important to ATP production, it is no object of active metabolic control.

The reaction *nuo* (NADH dehydrogenase I) obtains the fourth highest FC. It is one of two reactions which oxidize NADH to NAD and transfer the electrons to ubiquinone reducing it to ubiquinol. The free energy is utilized by *nuo* to pump four protons from cytosolic to periplasmic space generating proton-motive force. The other reaction *ndh* (NADH dehydrogenase II) fulfills the same function without pumping any protons. It obtains low FC.

The reaction *cyoABCD* (cytochrome *bo3* oxidase) obtains highest FC following *nuo*. The reaction *cyoABCD* converts ubiquinol back to ubiquinone and transfers the electrons to oxygen as the terminal electron acceptor which is reduced to water. Simultaneously, it pumps four protons from cytoplasmic to periplasmic space. The reaction *cydAB* (cytochrome *bd* oxidase) fulfills the same function while pumping two instead of four protons. As nitrate is available in the examined setting, the reaction *narGHI* can backup the function of *cydAB* and convert ubiquinol to ubiquinone while pumping two protons but utilizing nitrate as terminal electron acceptor instead of oxygen. The reaction *cydAB* obtains a low FC.

The other reactions with high FC are *fum*, *gltA*, *acnA* and *acnA_r2* which are all part of the tricarboxylic acid (TCA) cycle. The reducing equivalents NADH and NADPH are prevalently generated in the TCA cycle. NADPH is converted to NADH by the reaction *udhA*, such that it can be utilized for ATP production in the ETC. The reactions *fum*, *mdh*, *mqo*, *gltA*, *acnA*, *acnA_r2* can be short-circuited by the glyoxylate bypass utilizing *aceA* and *aceB*. This enables partial functioning also when *icd* or *sucABCD* are inactive. The reactions *mdh* and *mqo* obtain much lower FCs since each one backups the other's function in the TCA cycle.

Metabolic functions exhibit specific FCs as shown in the previous sections. We compare FCs of biomass, ATP and lactate production under aerobic conditions by utilization of Kendall's rank correlation on FC rankings obtained as described in the Methods section. The values are provided in Table 2.

**Table 2:** Pairwise correlation between functional centralities for different metabolic functions.

Function		O_2_-respiration	NO_3_-respiration	Fermentation
# 1	# 2	Kendall's *τ*	Kendall's *τ*	Kendall's *τ*
BM	ATP	0.426^*^	0.498^*^	0.59^*^
BM	LAC	0.167	0.09	0.168
ATP	LAC	−0.148	0.015	0.427^*^

Examined metabolic functions: biomass production (BM), ATP production (ATP), lactate production (LAC). Significant correlations 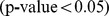 are marked by ^*^.

Related metabolic functions should show correlated FCs. The production of biomass is related to the production of ATP for two reasons. First, ATP is a major biomass component, which implies that maximization of biomass production necessitates high production of ATP. Second, ATP is the metabolism's major energy currency involved in many energy demanding reactions in central carbon metabolism. Therefore, maximization of biomass production requires high production of ATP to fuel the ATP demand of several biomass synthesizing reactions. The close relation between the two metabolic functions is reflected in a significant high correlation of the respective FCs (Table 2).

On the other hand, unrelated metabolic functions should show un- or lowly correlated FCs. Lactate production is largely unrelated to biomass production, since lactate is not a biomass component. Furthermore, it is a simple metabolic function compared to both, ATP and biomass production: it is produced and consumed only by a small number of reactions whereas the biomass component ATP is produced and consumed by multiple reactions all over central carbon metabolism. In line with this, we observe that biomass production as well as ATP production are not significantly correlated with lactate production with the exception of ATP production and lactate production under fermentative conditions (see Table 2). Under fermentative conditions, production of lactate as a major fermentation product is important for the production of ATP which is reflected in a large positive correlation between the two metabolic functions.

#### Dependence on environment

Environmental conditions shape metabolic states [78], [79] and should therefore be reflected in the structural metabolic control capabilities of individual reactions. *E. coli* is a facultative anaerobe bacterium which means that it is able to drive ATP production via aerobic respiration but also to respire anaerobically or even to switch to fermentation if oxygen is absent [17]. The respiratory state depends on the available terminal electron acceptors. If oxygen is available *E. coli* converts glucose to carbon dioxide for ATP production using oxygen as terminal electron acceptor in aerobic respiration. If nitrate is available and oxygen is not, *E. coli* switches to nitrate respiration which then is utilized as terminal electron acceptor instead. If exogenous terminal electron acceptors are unavailable, *E. coli* switches to fermentation. Aerobic respiration is the preferred mode of operation with oxygen having the highest redox potential followed by nitrate [8].

The structural metabolic control capabilities of individual reactions should be unaffected by the environmental conditions if these do not affect metabolic function. Exogenous terminal electron acceptors like oxygen or nitrate are needed for efficient ATP production from carbohydrates, as pointed out above. The metabolic function of lactate synthesis does not need any ATP generated by respiration: degradation of one molecule of glucose to pyruvate utilizing the phosphotransferase system *ptsGHI* actually consumes one molecule of ATP but produces four. Pyruvate then can be converted to lactate in one step utilizing lactate dehydrogenase *ldhA* without requiring any ATP. Hence, the maximum of lactate production does not depend upon the available terminal electron acceptor and, therefore, not on the examined environmental condition. In line with this, we observe that FCs for lactate production are perfectly correlated between environmental conditions (Table 3, the complete values obtained for FC can be found in Tables S5, S7 and S8).

**Table 3:** Pairwise correlation between functional centralities under different environmental conditions.

Env. condition		ATP production	LAC production
# 1	# 2	Kendall's *τ*	Kendall's *τ*
O_2_	NO_3_	0.686^*^	1^*^
O_2_	Ferm	−0.011	1^*^
Ferm	NO_3_	0.217^*^	1^*^

Environmental conditions: aerobic respiration (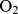), nitrate respiration (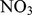) and fermentation (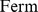). Significant correlations 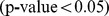 are marked by ^*^.

Environmental changes largely affecting the operation of a metabolic function should have significant impact on individual reactions' structural metabolic control capabilities. FCs for aerobic and nitrate respiration should be related, since the redox potential of nitrate is relatively large and its utilization theoretically enables respiring glucose completely to carbon dioxide (implying maximal production of reducing equivalents) [8], [17]. Indeed, we obtain a large correlation between the two environmental conditions (Table 3).

We find significant differences in FCs of the ETC, where the available terminal electron acceptors have effect (Figures S2 and S3; highest ranked reactions are given in Table 1, all ranks are shown in Table S4 and the complete values are given in Tables S6 and S9). The remaining reactions obtain similar FCs. The oxygen import *o2* as well as the cytochrome oxidases *cydAB* and *cyoABCD*, which obtain high FCs under aerobic conditions, cannot carry any flux under conditions of nitrate respiration. They obtain zero FC. Instead, the reactions with highest FC are the nitrate import *no3*, nitrate reductase *narGHI*, which oxidizes ubiquinol while pumping two electrons from cytosolic to periplasmic space using nitrate as terminal electron acceptor, and *no2* which exports the resulting nitrite. These three reactions exhibit low FCs under aerobic conditions.

FCs are substantially different under fermentative conditions (Figure S4; highest ranked reactions are given in Table 1, all ranks are shown in Table S4 and the complete values are given in Table S10). Here, no exogenous terminal electron acceptors are available, which implies that there is no possibility to oxidize NADH in the ETC to pump protons. Therefore, ATP production is drastically decreased under this condition. The reactions with highest FC follow the path leading from glucose import by *ptsGHI* down glycolysis to pyruvate. This path already enables a maximum production of three ATP achievable under fermentative conditions. The export of ATP *maint* also obtains high FC. The remaining reactions with high FC lead from pyruvate to acetate, ethanol, formate and lactate. Excretion of one or more of these products is mandatory for ATP production, since without oxygen or nitrate as terminal electron acceptors, it is not possible to oxidize glucose fully to carbon dioxide. All of these metabolites are major fermentation products of *E. coli* [68].

Altogether, we observe that FCs and, therefore, structural metabolic control capabilities, change largely between respiratory conditions and fermentation. FCs shift from TCA to glycolysis, from the export of carbon dioxide to export of fermentation products and away from the ETC. The ETC is central to efficient ATP production. Apparently, its manipulation must have serious impact. In the case of fermentation, oxygen and nitrate are not available and the respective reactions of the ETC driven by these electron acceptors cannot be utilized. Therefore, control of ETC must have far less impact on ATP production in that case.

### Application to data

Stelling *et al.* [45] have shown for *E. coli* that changes in CEFs with respect to growth on different substrates are correlated to changes in messenger RNA levels which predominantly account for physiological changes on longer timescales. To compare the capability to predict changes in transcript levels, we repeat the respective analysis [45] and calculate CEFs and FCs for the substrates acetate and glucose and relate the differences to transcript data [80].

CEFs are calculated for biomass and ATP production and averaged with weighting by maximal yield as described in the Methods section. Accordingly, we calculate FCs for biomass and ATP production and average the normalized FCs.

**Figure 5:**
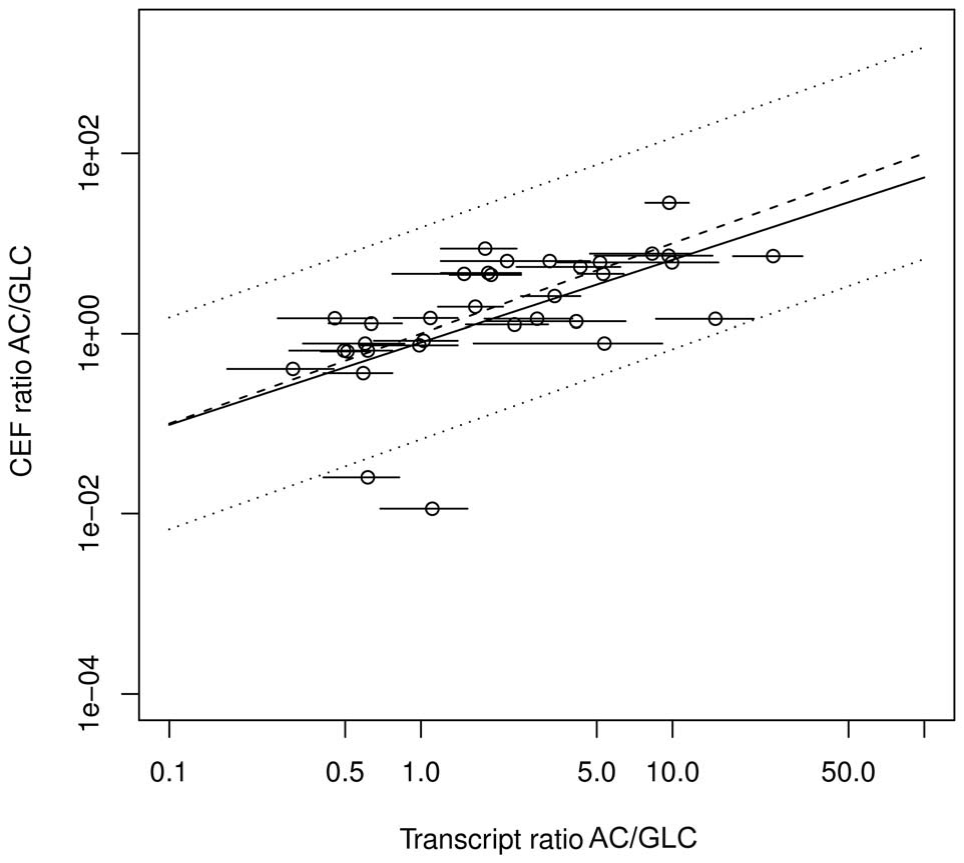
Prediction of gene expression patterns by control-effective fluxes (CEFs). Calculated ratios between transcript levels during exponential growth on glucose (GLC) and growth on acetate (AC) under conditions of aerobic respiration based on CEFs versus experimentally determined transcript ratios. Lines indicate 95% confidence intervals for experimental data (horizontal lines), linear regression (solid line), perfect match (dashed line) and two-fold deviation (dotted line).

**Figure 6:**
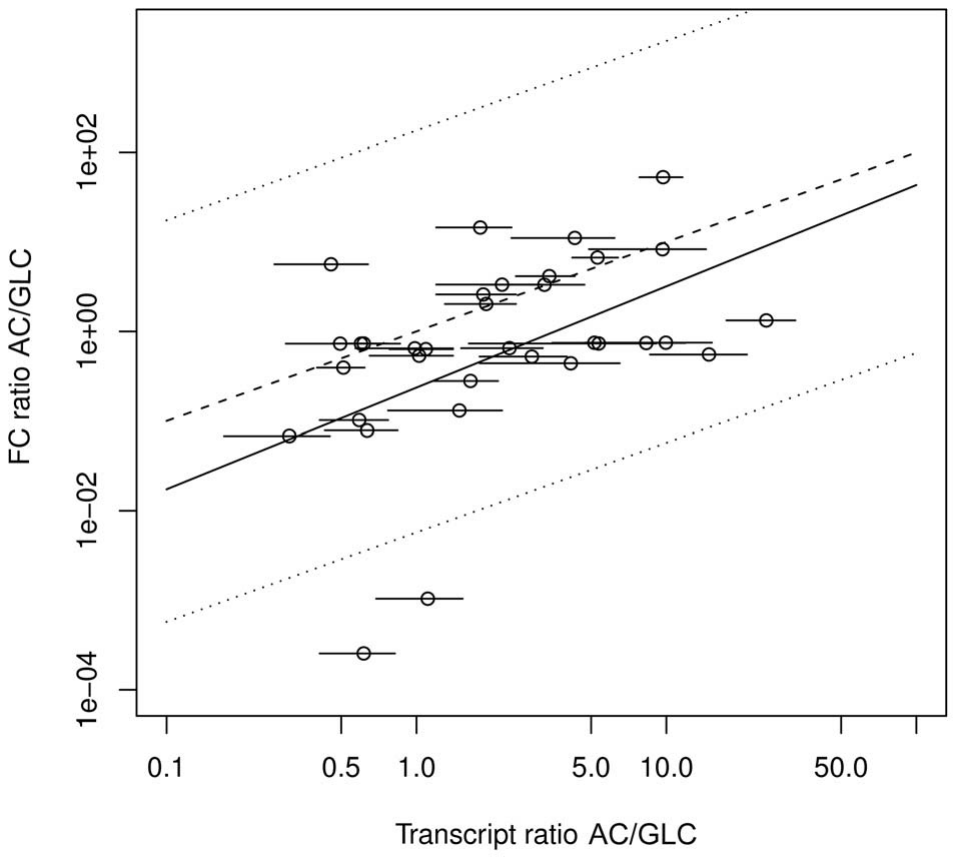
Prediction of gene expression patterns by functional centralities (FCs). Calculated ratios between transcript levels during exponential growth on glucose (GLC) and growth on acetate (AC) under conditions of aerobic respiration based on FCs versus experimentally determined transcript ratios. Lines indicate 95% confidence intervals for experimental data (horizontal lines), linear regression (solid line), perfect match (dashed line) and two-fold deviation (dotted line).

Graphical analysis exhibits good agreement for the ratio of transcript levels with the ratio of CEFs (Figure 5) and also reasonably good agreement with the ratio of FCs (Figure 6).

We find for CEFs as well as for FCs three reactions exceeding two-fold deviation of the regression line: *pfk*, *ack* and *pta*. The reactions *ack* and *pta* account for acetate production and should not have large relevance for ATP and biomass production under aerobic conditions for either utilization of acetate or of glucose. In line with this, transcript levels differ only slightly for the two substrates. In contrast, CEFs and FCs change largely between the conditions. This shift to larger values for growth on glucose is reasonable: while utilization of these reactions has no advantage for growth on acetate (because acetate is imported and therefore does not have to be produced), acetate production can make sense for growth on glucose, *e.g.*, in the course of fermentation to adapt to fluctuations of the availability of exogenous electron acceptors. CEFs and FCs suggest an upregulation of *pfk*, which is part of glycolysis, upon switch from acetate to glucose. The reason that transcript levels of *pfk* change only slightly might be a strategy of anticipation, enabling quick response if glucose, the preferred carbon source, is available again. The discrepancies between predictions based on structural metabolic control capabilities and experimental data may indicate that realization of control through manipulation of potential control sites is not necessarily unique.

Kendall rank correlation of the average of transcript ratios with CEFs (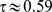, 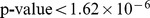) is larger than with FCs (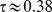, 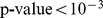). The reason may be that CEFs, in contrast to FCs, incorporate an efficiency criteria aiming at the minimization of total flux. Minimal metabolic investment might be another important factor in the realization of metabolic control, which has also been proposed as a design principle of metabolism elsewhere [81], [82].

Changes of CEFs between conditions show larger association with transcript ratios compared to FCs. However, it is unclear how the shift in transcript expressions affect metabolic flux, which finally is the target of FCs.

We further tested the hypothesis of an association between structural metabolic control capabilities and the number of transcription factors affecting individual enzyme catalyzed reactions. CEFs as well as FCs are dependent on the environment and on the metabolic function. As the number of transcription factors is a static feature, we expect an association only in a metabolic function that is vital to the survival of *E. coli*. The composition of biomass may change with conditions, *e.g.*, with growth rate [83]. Therefore, we choose ATP production, whose control is vital and which does not exhibit a problem of likely condition-dependent composition. We examine consumption of glucose under conditions of aerobic respiration, which is the environment with largest scope in controlling ATP production. The number of transcription factors associated to individual enzymes where derived from RegulonDB [84]. We obtain a Kendall rank correlation coefficient of 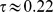 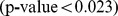 in the case of CEFs and 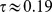 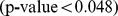 in the case of FCs. Both measures yield a comparable significant association between number of transcription factors affecting reactions and their ranking in the respective measure of structural metabolic control. We conclude that both measures capture to some degree the feature of regulatory effectors. However, the association is relatively small. This is in line with our expectation as metabolic control is a dynamic feature depending on environment and physiological state in contrast to the number of transcription factors.

## Discussion

FC has proven to be suitable to identify potential sites of control using structural modeling. Knockout of reactions chosen with respect to FCs have shown the largest effect on the metabolic function of biomass production compared to the other measures and uniform sampling as a reference in the multiple knockout study. The results are stable across environmental conditions. Furthermore, the structural metabolic control capabilities, determined by FC, have shown to be shaped by environmental conditions as well as by metabolic function. This implies that the quality of predictions for complex composite metabolic functions, such as biomass production, depends on the accuracy of the experimental data the composition is based on. The composition of biomass may also change with conditions, *e.g.*, with growth rate [83].

CEFs have been introduced to link EFM properties to regulation patterns of transcripts and have shown good capability to identify potential sites of control in the multiple knockout study. Strikingly, while FCs and CEFs have shown the best results among the examined measures, both are only weakly correlated. FCs exhibit by far larger correlation to EFMs than to CEFs (Table 4). This suggests, that both measures capture important but different aspects of (structural) metabolic control. The most obvious distinction is that CEFs, in contrast to FCs, integrate weighting of minimal metabolic expenditures to realize a specific flux with respect to a metabolic function. A modification of FCs to incorporate such weighting might improve its predictive power and is described below. While deciphering the differences of the two approaches is difficult due to their complexity, it promises further valuable insights. The two approaches demonstrate that the complex nature of metabolic control is hard to capture already in the strongly simplified perspective of structural metabolic control.

**Table 4:** Pairwise correlation between measures of structural metabolic control capabilities for the metabolic function of biomass production.

Measure		O_2_-respiration	NO_3_-respiration	Fermentation
# 1	# 2	Kendall's *τ*	Kendall's *τ*	Kendall's *τ*
FC	FCA	0.107	0.228^*^	−0.15
FC	EFM	0.45^*^	0.348^*^	0.507^*^
FC	EFM_yield_	0.443^*^	0.334^*^	0.523^*^
FC	CEF	0.184^*^	0.229^*^	0.379^*^

Pairwise correlation of functional centralities (FC), coupling degrees (FCA), reaction participations in biomass producing EFMs unweighted (EFM) and weighted by biomass yield (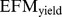) as well as of control-effective fluxes (CEFs). Significant correlations 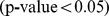 are marked by ^*^.

Pathway yield has a significant impact on the structural metabolic control capabilities of participating reactions. Weighting by yield improved the identification of potential sites of control in the case of reaction participation and is also incorporated in the efficiency weighting of CEFs and implicitly in the calculation of FCs.

The compliance with transcript data was better for CEFs than for FCs. However, it is difficult to determine how the changes of transcript expressions affect metabolic flux. A potential reason for the difference could be the aforementioned preference of CEFs for short pathways accounting for a minimization of cellular resource expenditures. FCs could be modified to incorporate such a criteria by utilization of quadratic programming. We propose two procedures: (i) maximizing metabolic function per total flux, and, (ii) maximizing metabolic function and subsequently minimizing total flux with fixed optimal flux of the metabolic function, then utilizing the ratio of the two optimization outcomes. On the other hand, the association of CEFs and FCs, respectively, with the number of transcription factors acting on the reaction catalyzing enzymes indicated comparable potential of the two measures to explain such static regulatory features, although the association was minor.

The flux coupling degree of reactions has shown to be largely unable to predict potential sites of control in the multiple knockout study. The reason most likely is that flux coupling analysis quantifies only pairwise interactions of reactions and that in systems with high flexibility fluxes are widely disentangled. This highlights that structural metabolic control capabilities predominantly arise from interactions on the system level which is further corroborated by the results from the remaining measures. All of which integrate system level interactions and exhibit significant qualification to identify potential sites of control.

The estimation of FCs is based on EFM enumeration, which limits the accessible network size. The combination of our algorithm with sampling of EFMs or enumeration of a specific subset could enable the approximation of FCs for up to genome-scale metabolic networks. We describe three approaches in the following.

EFMs with low numbers of participating reactions have higher weights in the sampling procedure of the FC estimation than long ones (see Methods section). Therefore, short EFMs are expected to dominate the sampling procedure. De Figueiredo *et al.* have described an algorithm to compute the k-shortest EFMs which could be utilized with this respect [85]. However, the accuracy would eventually depend on the (unknown) length distribution of EFMs and the bias introduced by this approach would be difficult to analytically specify.

Another possibility is to determine the specific subset of EFMs with respect to a certain metabolic function and in accordance with environmental conditions which would enable estimation of the corresponding FCs. Kaleta *et al.* combine linear programming with a genetic algorithm [86] to enable targeted sampling of EFMs in genome-scale metabolic networks. The authors argue to enumerate a significant portion of EFMs capable of the synthesis of specific amino acids in a genome-scale model of *Corynebacterium glutamicum* comprising 641 reactions growing under aerobic conditions with glucose as substrate. If a large fraction of functional EFMs can be determined, it might enable estimation of FCs with minor bias. Nevertheless, such computation for networks comprising several thousands of reactions is likely to be too demanding regarding computational resources and time consumption.

Unbiased sampling of EFMs, meaning sampling which resembles the length distribution of the full set of EFMs, would facilitate unbiased estimation of FCs (see Methods section). This seems to be most promising to enable estimation of FCs on genome-scale. Machado *et al.* have described an algorithm to draw a sample from the full set of EFMs which they claim to be unbiased as long as the sample size is large [87]. While this approach is in principal applicable to genome-scale networks, its current implementation is presumably not fast enough to obtain a sample of sufficient size in reasonable time. Furthermore, it is unclear if a sample size guaranteeing unbiased sampling is accessible for genome-scale networks. A promising approach to sample EFMs has recently been described by Tabe-Bordbar and Marashi who utilize an algorithm based on linear programming [88]. Their approach is applicable to genome-scale metabolic networks and is claimed to be unbiased irrespective of the sample size. While we present an accurate derivation of the estimation errors of FCs in this study, it is unclear at the moment how these errors are to be determined when utilizing a representative sample of EFMs instead of the full functional set.

In addition, it is equally important to apply preprocessing steps aimed at reducing the complexity of the enumeration problem. Jol *et al.* employed a preprocessing strategy to reduce the complexity of the EFM calculation by checking reversibilities of reactions by flux variability analysis [66] in advance and by incorporation of metabolomics data to further restrict reaction reversibilities to physiological ones. Furthermore, they integrated the obtained EFMs with metabolomics data to reduce the set of EFMs to comprise only thermodynamically feasible EFMs. While the latter does not reduce the complexity of EFM enumeration, it is expected to increase the predictive power of FCs.

Eventually, developments with respect to hardware and software are underway, together exhibiting the potential to enable EFM enumeration for considerably larger networks than possible at present [89].

### Concluding remarks

In this study, we have presented an algorithm to estimate functional centralities (FCs) for large metabolic networks. Moreover, we have demonstrated the potential of FCs to determine structural metabolic control capabilities of individual reactions. FC accomplishes this by explicitly integrating the potential interactions of reactions with the remaining system, covering interactions with individual reactions, with pathways and with subnetworks. The study exemplifies that important properties of metabolic control can be accessed from structural information of a metabolic network.

The results of FBA can be improved by integration of additional information about physiological flux boundaries obtained from high-throughput measurements [90]. This may, in turn, also improve the predictions obtained by FC. Moreover, while FC cannot directly integrate kinetic parameters, it is possible to modify the framework to incorporate extensions of FBA accounting for, *e.g.*, molecular crowding [91] or membrane economics [92].

Currently, the most established measure to capture control in metabolic networks are flux control coefficients (FCCs) of MCA. Besides the discussed drawbacks of this approach, we want to highlight that FC captures the potential effect of the manipulation of multiple reactions, while FCCs only describe the effect of individual manipulations.

FC enables the design of knockout and overexpression strategies, taking into account the complexity of metabolic control. For instance, FC can be utilized to suggest targets of metabolic engineering to decrease the production of an unwanted byproduct which limits the yield of a desired product. To this end, the two production processes are defined as metabolic functions and FCs are calculated individually. Those reactions which are functionally central for the production of the unwanted byproduct but not for the production of the desired product are suggested as knockout candidates. A similar strategy could be applied in the identification of drug targets.

We have shown that FCs are shaped by environmental conditions and the physiology of metabolic functions. It would be of further interest to examine if an association between FCs and synthesis costs of the corresponding enzymes [73] exists, as it is reported for utilization of EFMs and their resource requirements under certain conditions [93]. Such analysis could also provide insights into how a manipulation pattern of potential sites of control, which is not necessarily unique, might be chosen.

We have formulated the calculation of estimated FCs such that the calculation can be performed for only a subset of reactions. While an application would have been out of scope of this study, it provides the means to examine control exerted by a subset of reactions on an enclosing system. In that case, elementary flux pattern calculation [94] could serve to determine the elemental coalitions, which would even enable the examination of control for the case that the enclosing system is of genome scale. This may enable, *e.g.*, the calculation of structural metabolic control capabilities of the reactions of the central carbon metabolism on biomass production of the genome-scale network.

FCs do quantify the relevance of reactions to a metabolic function considering all possible interactions, but they do not quantify the interactions themselves. Grabisch and Roubens have defined a measure extending the classical Shapley value to capture interactions among any set of elements [95]. This approach has been utilized to determine interactions between any two elements to examine perturbation experiments in the setting of neuroscience [96] and gene regulation [97]. While extension of FC in accordance to this measure is appealing, it is unclear if the approach can be modified to apply to the restriction to functional subnetworks only. Furthermore, it is ambiguous if the accompanying increase in computational demands can be met.

Linking metabolism and regulatory events is inherently difficult and by far from being completely characterized. Regulatory mechanisms act on multiple levels, such as transcriptional regulation, post-translational modifications and metabolite-protein interactions; all of which ultimately exerting control on metabolic flux [98], [99]. Functional centralities have shown to be a prospective approach for deeper understanding of metabolic control. To analyze the association between regulatory events and potential sites of control, sophisticated and targeted experiments are needed.

## Methods

### Flux balance analysis

Flux balance analysis (FBA) is a structural modeling framework developed to characterize the synthesizing capabilities of metabolic networks at steady state [67]. A metabolic network consists of 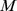 metabolites 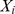 (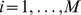) and 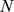 reactions. The change of the concentration 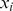 of a metabolite 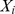 can be described as 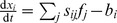, where 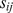 is the stoichiometric coefficient associated with the flux 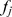 through reaction 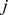 and 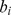 is the net transport flux of metabolite 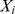. The mass conservation relation under steady-state conditions, *i.e.*, 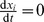, results in the following expression:
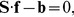
(3)where 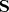 is the stoichiometric matrix (with 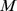 rows and 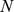 columns), 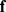 is the vector of metabolic fluxes of the 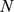 reactions and 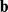 is the vector representing consumption/production fluxes of the 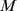 metabolites. The consumption/production fluxes are set to zero for internal metabolites. In contrast, external metabolites constitute an interface to the environment and do not have to obey the steady-state condition. A metabolic flux crossing the system boundary is normally realized by a transporter reaction which converts an internal metabolite into an external metabolite. Constraints on the fluxes of the transporter reactions importing or exporting metabolites across the system boundary are utilized to establish environmental conditions, *e.g.*, determining the availability of nutrients. As the system of equations described in (3) is usually under-determined (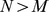), there exist multiple solutions corresponding to feasible flux distributions, each representing a particular metabolic state (with respect to fluxes) satisfying the stoichiometric constraints. The question usually addressed by FBA is that of determining which of the feasible metabolic states is manifested in the studied metabolic network.

FBA relies on the assumption that the metabolic system exhibits a state that is optimal with respect to some objective. Usually, the objective is expressed as a linear combination of fluxes in 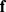, which leads to a linear programming problem:
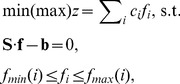
(4)with 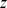 representing the phenotypic property to be optimized, and 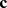 being a vector of coefficients quantifying the contribution of each flux to this objective. The bounds 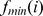 and 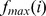, represent constraints on the fluxes, *i.e.*, the minimum and maximum values for the fluxes and, thus, determine reaction reversibility.

A common choice for the objective function is the maximization of biomass production, which allows a wide range of predictions consistent with experimental observations for simple model organisms [35]–[37]. This function could be employed for environments with nutrient excess. Another possible choice is minimization of 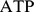 production which can be used to determine the conditions for energy efficiency and minimization of nutrient uptake, and is usually applied in modeling the case of nutrient scarcity (an overview of objective functions can be found in [22]).

We identify a metabolic function with the objective function 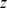 and calculate the synthesizing capacity of the metabolic function by maximizing the objective. It should be noted that this does not necessarily coincide with the objective responsible for a specific phenotype.

### Functional centrality

Functional centrality (FC) aims at assigning individual reactions of a metabolic network their contribution to a metabolic function [42]. Technically, it combines a solution concept from cooperative game theory with FBA.

A cooperative game is defined by a set of players 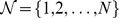 and a characteristic function 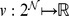 with 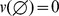 which assigns every subset 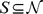 of the player set the worth it generates by cooperation. To find a *fair* and *unique* distribution of the cooperatively gained worth 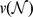 amongst the set of players, the solution concept of the *Shapley value* [43] has been introduced. The Shapley value is axiomatically founded and associates with every game 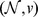 with *transferable utility*, *i.e.*, without restrictions on the division of 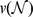, a unique and fair payoff vector. Thereby, the property of fairness of this solution is implied by the required axioms. The Shapley value of a single player 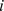 is given by the weighted sum of the player's contribution to all subsets of players.

We identify 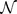 with the set (or a subset) of reactions forming the metabolic network and 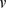 with the optimal value of the objective function determined by FBA for a subnetwork formed by the members of 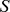 (if not all reactions of the network are considered, the subnetwork is formed by the members of 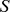 and the not considered reactions; in that case the objective function has to be formulated such that 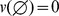 is valid). An extensive derivation is to be found in [42].

The classical Shapley value incorporates all 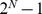 subsets of 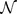. With respect to metabolic networks, some of these subsets correspond to nonfunctional subnetworks which are incapable of carrying nonzero flux with respect to the objective function. There are two possibilities to address the issue: (i) assigning zero worth to the nonfunctional subnetworks, and (ii) excluding the nonfunctional subnetworks from the calculation of the Shapley value. FCs make use of a modified version of the Shapley value [44] to exclude the nonfunctional subnetworks and account only for the functional ones. It has been shown that exclusion of nonfunctional subnetworks is superior for determining reactions' contribution to metabolic function [42].

To introduce the modified version of the Shapley value, as proposed by Aguilera *et al.* [44], let 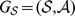 be a directed graph. The set of nodes 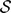 encompasses all subsets 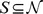 which correspond to functional subnetworks plus the empty set 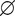. The set of arcs 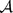 consists of all 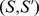 with 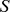
⊆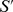 for which it holds that there exists no 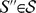 with 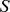
⊆
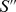
⊆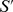. The graph 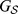 is induced by inclusion on 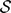 and is equivalent to a Hasse diagram [100]. In 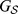, every path from the empty set to the set 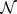, encompassing all (considered) reactions, represents one possibility to add players successively in such a way that the corresponding subnetworks belong to the family of functional subnetworks. These paths are called *maximal chains*. The graph 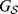 is called *regular*, if all maximal chains of 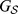 have equal length, otherwise it is called *irregular*. The calculation of the modified Shapley value is as follows:

Let 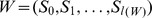 with 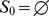 and 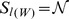 be a maximal chain for inclusion in 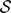, implying 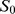
⊆
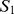
⊆
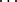
⊆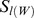. Then, the contribution of player 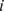 in maximal chain 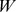 is given by
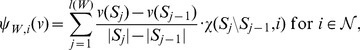
(5)

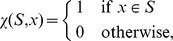
with 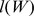 being the length of the maximal chain 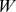. Let the set of all maximal chains be denoted by 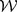. If 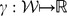 satisfies
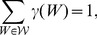
(6)then the weighted sum of contributions over all maximal chains
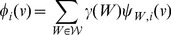
(7)defines the Shapley value of player 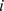 for arbitrary families of subsets. By providing an appropriate definition for the weighting factor 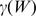, the value becomes uniquely determined [44]. In the calculation of FCs, we weight the maximal chains equally:
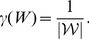
(8)This facilitates the error calculation of estimated functional centralities and results in only subtle differences to weighting by chain length [42] (data not shown). The axioms defining the modified Shapley value with respect to FC are given in the Supplementary Text S1.

### Estimation of functional centralities

The exact calculation of FCs is limited to small metabolic networks. The accessible network size, in terms of number of reactions, depends on computer hardware and network structure. The reason is the combinatorial explosion of the number of functional subnetworks and the number of corresponding maximal chains. Castro *et al.* describe an approach to estimate classical Shapley values, considering all subsets of 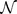, of large systems utilizing Monte Carlo sampling of maximal chains [101]. We modify this approach to estimate FCs which, in contrast, are based on a restricted set of subsets.

We first present the Monte Carlo algorithm, followed by explaining the sampling procedure. At the end of the section, we derive an approximation of the error of the calculated FCs by utilizing resampling, once the calculation has finished.

#### Algorithm for estimating functional centrality

FCs are calculated as the average of contributions of individual reactions in all maximal chains. Therefore, FCs can be estimated by the average contribution in a random sample of maximal chains, whereby each maximal chain is chosen with equal probability. In the approach of Castro *et al.* [101], the classical Shapley value is estimated. In that case, all subsets of 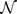 are considered (which would correspond to all subnetworks being functional in the case of FCs). Therefore, the graph 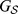 is regular and each of its maximal chains corresponds to one specific permutation of 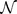. Then a set of random permutations of 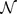 can be utilized for the estimation.

We modify the approach of Castro *et al.* to estimate FCs. In our case, the structure of 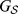 is irregular and depends on the metabolic network, as well as on the environmental conditions and the metabolic function. This renders the choice of maximal chains more complicated. The detailed algorithm is presented as pseudocode in Table 5. It should be noted that the estimator of FCs obtained by our algorithm is unbiased (meaning the expectation of the estimator equals the true value) and yields a division of 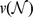 (in accordance with the requirement of the *efficiency axiom* given in the Supplementary Text S1). The proof of both properties is straightforward.

**Table 5:** Algorithm for estimation of functional centralities.

**Begin** **Set** 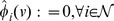 For ***k*** = **1 to** ***samplesize*** do **Choose elemental coalition** *E_k_* **with probability** *P*(*E_k_*) **Set *S*_1_: = *E_k_*** **Calculate *v*(*E_k_*)** **Divide *v*(*E_k_*) equally among the participating reactions 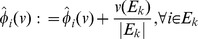 ** **Take 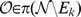 with probability 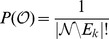 ** For ***m*** ** = 2 **to 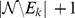 do **Set ** 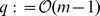 **Set** 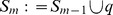 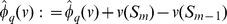 End End 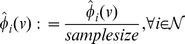 **End**

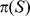
 denotes the set of all permutations of a set 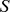 and 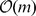 the 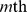 element of the set 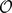.

The sampling of maximal chains is divided into two parts: (i) the choice of the first nonempty element of the maximal chain, which we call *elemental coalition*, and (ii) the choice of the order of adding the remaining reactions to the elemental coalition.

The elemental coalitions 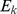 denote reaction sets corresponding to minimal functional subnetworks capable of the examined metabolic function under specific environmental conditions. The elemental coalitions 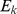 are minimal in the sense that diversion of any reaction from 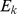 turns the subnetwork to nonfunctional. Therefore, in the case considering all reactions of a metabolic network, each elemental coalition 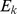 is equivalent to a reaction set corresponding to an EFM. Each maximal chain starts with the empty set followed by an elemental coalition 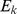 as first nonempty set.

The reactions' contributions in all sampled maximal chains are stored. This enables approximating the population variance of contributions of the individual reactions and, therefore, to assess the error of estimated FCs, as described at the end of the section.

#### Sampling of maximal chains

All maximal chains have the same probability to be chosen, but the graph 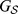, representing the structure of accessible maximal chains, is irregular and, therefore, the set of maximal chains is *a priori* unknown. In the following, we describe how to determine the structure of 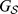 and how to obtain an unbiased sample of the full set of maximal chains.

In principal, the graph 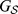 can be reconstructed from the set of functional subnetworks which then have to be determined in advance [42]. Due to combinatorial explosion this is impractical for large systems. However, we can exploit the characteristics of the computation of FCs to determine the structure of 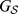 without checking every subnetwork upon whether it is functional or nonfunctional. To this end, we utilize the EFMs of the network, which constitute minimal subnetworks yielding nonzero flux at steady state and are, hence, closely related to the elemental coalitions. The EFMs can be obtained by utilizing available software packages like *EFMtool* [102].

In the case considering all reactions of the metabolic network, the elemental coalitions correspond to the set of functional EFMs, *i.e.*, the ones capable of the examined metabolic function in accordance with environmental conditions. If only a subset of reactions of the metabolic network is examined, additional operations have to be performed on the set of EFMs to obtain the set of elemental coalitions which are described below. Since inclusion of any player to a functional subnetwork likewise results in a functional subnetwork [42], the structure of 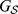 (which is induced by inclusion) is fully determined by the set of elemental coalitions.

The investigation of only a subset of reactions could have several reasons, *e.g.*, reactions importing nutrients or exporting products, respectively, may be assumed to be always active. Another possible application is investigating FCs of only a subsystem to infer control of the subsystem's components on the enclosing system.

When only a subset of the reactions of a metabolic network is considered, it occurs that only some of the reactions of EFMs correspond to considered reactions. Then functional EFMs may not correspond to elemental coalitions (an example is shown in Figure S5). To determine the elemental coalitions in that case, the set of functional EFMs is first represented by a matrix 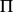, with 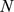 columns corresponding to the 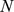 reactions such that the entries of any row give the fluxes through the respective reactions of a functional EFM. (Since we are only interested in a reaction being active or inactive, the entries can be converted to boolean values in order to reduce computational demands in the further procedure.) The set of elemental coalitions then is obtained by: (i) removing all columns of 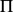 corresponding to the unconsidered reactions and (ii) reducing the resulting matrix 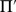 to a matrix 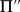 containing only rows such that the set of indices of the nonzero elements of any row is not a subset of the set of indices of the nonzero elements of any other row (for any row 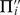 of 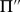, let 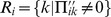; for any row 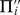 of 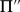 there exists no row 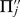 with 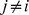 in 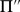, *s.t.* 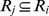). The complexity of the matrix reduction scales quadratically with the number of EFMs. The elemental coalitions are then composed of the reactions corresponding to the nonzero entries of the rows of matrix 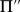.

As mentioned above, the addition of any reaction to a functional subnetwork also results in a functional subnetwork. Hence, the number of maximal chains 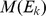 starting from a specific elemental coalition 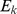 is the factorial of the number of the remaining reactions:
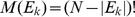
(9)The total number of maximal chains then is the sum of the number of maximal chains for all elemental coalitions,
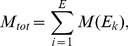
(10)with 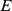 being the total number of elemental coalitions. We can then calculate the probability of choosing a maximal chain starting with a specific elemental coalition 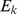:
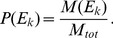
(11)Each maximal chain starting with 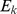 describes a sequence of adding the remaining reactions 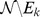, such that all permutations of the remaining reactions are covered. Then the probability to choose a maximal chain from the set of maximal chains starting with 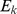 is 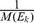.

Together this enables drawing a truly random sample of maximal chains as described in the algorithm presented in Table 5.

### Error calculation of estimated functional centrality

The error of the estimated FCs depends on the utilized sample size. In [101], the authors give a description on determining a sample size that guarantees a specific upper boundary of the error. Instead, we describe how resampling can be utilized to obtain a good approximation of the real error, once the calculation has finished. (We observe that the real error is by far smaller than its upper bound, data not shown.) Since it is impractical to define a stopping criteria, the error has to be obtained *a posteriori*.

Utilizing a sample of sufficient size 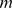 guarantees that the error of the estimated Shapley value 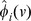 of reaction 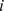 is smaller than 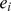 with a probability larger than 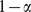: from the central limit theorem follows 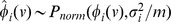, where 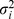 is the population variance of the contributions of reaction 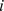 and 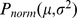 the normal distribution with expectation 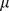 and variance 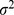. Hence, if for the sample size
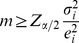
(12)holds, then
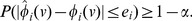
(13)With 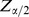 being the value, such that 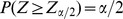, 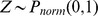. For a given sample size the error 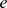 then is
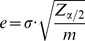
(14)


In this study, we have chosen 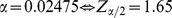. This guarantees that the error is accurate with probability larger 97.5%. We utilize a sample size of 200,000, which has shown a good balance between accuracy and computational demand in this study. It should be noted, that the error calculation is irrespective of the number of maximal chains due to the implications of the central limit theorem. An approximation of the population variance is given in the next section.

Special attention has to be paid to reactions which obtain zero marginal contribution in all samples, which could falsely lead to the conclusion of zero error. In that case, we assign the error that would be obtained for a single nonzero contribution with the value of the largest contribution found in the sampling process.

#### Variance estimation

Resampling of the recorded contributions of individual reactions per sample enables determination of the accuracy of the sample variance as an estimator for the population variance. To this end, we utilize the Jackknife-based Tukey's formula [103] which has been introduced to estimate the variance of a sample statistic, in our case the variance of the sample variance. The Jackknife resampling is based on calculating a statistic of a dataset with 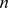 datapoints 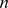 times, each time with exactly one datapoint having been diverted from the dataset.

Tukey's formula to estimate the variance of a statistic 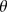 is
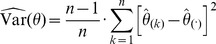
(15)with 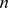 being the number of samples, 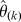 being the Jackknife statistic of the sample with datapoint 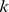 being excluded from the dataset and 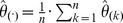 being the mean of the Jackknife statistics 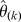. The Jackknife variance estimate tends to be conservative in the sense that it overestimates the true variance [103].

In our case, the statistic is the variance of estimated FCs 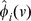. Then 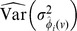 denotes the Jackknife estimate of the variance of 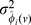. We utilize 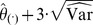 as an estimate of the variance of 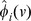, such that the probability to underestimate the variance is below 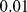 (under the assumption that Tukey's formula does not underestimate the variance of the statistic).

#### Rank calculation

The presented FCs are estimations and, therefore, the ranks of individual reactions might be indistinguishable with respect to their errors. To rank the reactions properly, we first sort the reactions according to FCs in descending order. If the sum of the errors of two successive ordered reactions is larger than the absolute difference of the reactions' FCs, we declare them indistinguishable and assign them the same rank. By this procedure, it may occur that a set of reactions is declared indistinguishable while the first reaction actually is distinguishable from a sequent reaction at the end of the set. The described rank calculation does incorporate less information than availabe but is unbiased.

### Control-effective fluxes

Control-effective fluxes (CEFs) are originally defined by *efficiencies* 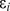 of the individual EFMs 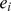 with respect to a substrate 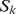 and the production of biomass (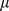) and ATP (EFMs are normalized by substrate uptake in advance) [45]:
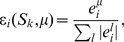
(16)

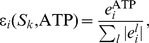
(17)whereby 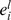 denotes the flux through reaction 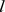 in the EFM 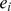. The CEFs 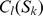 are then given by
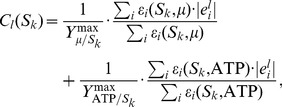
(18)with 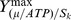 being the maximum yield of biomass or ATP production, respectively, for substrate 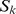. In the case examining a specific metabolic function as it is the case in the Monte Carlo multiple knockout study, the calculation of CEFs is restricted to this metabolic function. In the multiple knockout study, we examine biomass production as metabolic function and glucose (GLC) as substrate. In that case, we have to divert the term accounting for ATP production. Then CEFs reduce to:
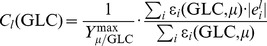
(19)


### Model

We utilize a model of central carbon metabolism of *E. coli* published by Schütz *et al.* [22]. The model comprises 74 unique reactions (given in Table S11), whereof 14 are transporters, and 61 metabolites (given in Table S12), whereof 47 metabolites are internal and, therefore, their concentrations have to obey the steady-state assumption. The metabolic network is able to import acetate, ethanol, glucose, nitrate and oxygen and to export acetate, ATP, biomass, carbon dioxide, ethanol, formate, lactate, nitrite, pyruvate and sucrose. Import of ethanol is disabled in the examined settings. Acetate import is disabled for growth on glucose and vice versa. Since ADP is an external metabolite in this model, the export of ATP does account for ATP hydrolysis rather than ATP *de novo* synthesis. The biomass export reaction comprises all metabolites from central carbon metabolism in appropiate ratios to support growth. The sum of glucose import by the reactions *mglABC* and *ptsGHI* has been constrained from above arbitrarily by one. Isozymes in the model were deleted since they add multiple layers of combinatorial complexity and, moreover, would bias FCs. An upper bound of one has also been utilized for acetate import in the calculation of FCs with acetate as a substrate. We examine three environmental conditions (following the study of Schütz *et al.*): (i) *aerobic respiration*, with no further restrictions (which implies nitrate being available as alternative terminal electron acceptor), (ii) *nitrate respiration*, anaerobic growth in the presence of nitrate (no oxygen import), (iii) *fermentation*, anaerobic growth without nitrate as electron acceptor (no oxygen and no nitrogen import). The utilized objective functions are the fluxes through the reactions: (i) *maint* (ATP production), (ii) *biomass* (biomass production), (iii) *lac* (lactate production).

## Supporting Information

Figure S1 **Functional centralities (FCs) for lactate production under conditions of aerobic respiration.** Thickness of arrows corresponds to FCs in the central carbon metabolism of *E. coli*.
(PDF)

Figure S2 **Functional centralities (FCs) for ATP production under conditions of aerobic respiration.** Thickness of arrows corresponds to FCs in the central carbon metabolism of *E. coli*.
(PDF)

Figure S3 **Functional centralities (FCs) for ATP production under conditions of nitrate respiration.** Thickness of arrows corresponds to FCs in the central carbon metabolism of *E. coli*.
(PDF)

Figure S4 **Functional centralities (FCs) for ATP production under conditions of fermentation.** Thickness of arrows corresponds to FCs in the central carbon metabolism of *E. coli*.
(PDF)

Figure S5
**Elemental coalitions with and without consideration of transporters.** Example network containing two EFMs. All considered reactions are shown in black, the blue dashed box marks an elemental coalition. **A** and **B** show the case considering transporters; the elemental coalitions equal the reaction sets of the two EFMs 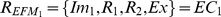 and 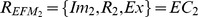. **C** and **D** show the case not considering transporters; the reaction set of the EFMs are reduced to 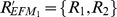 and 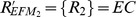, which is the only elemental coalition, since 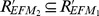.(PDF)

Table S1 **Normalized functional centralities for the metabolic function of biomass production under conditions of aerobic respiration (sample size 200,000).**
(PDF)

Table S2 **Normalized functional centralities for the metabolic function of biomass production under conditions of nitrate respiration (sample size 200,000).**
(PDF)

Table S3 **Normalized functional centralities for the metabolic function of biomass production under conditions of fermentation (sample size 200,000).**
(PDF)

Table S4
**Reaction ranks according to functional centrality (FC).** The table shows FCs for the metabolic function of lactate production (LAC) under conditions of aerobic respiration (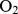), and ATP production (ATP) under conditions of aerobic respiration (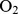), nitrate respiration (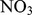) and fermentation (Ferm). Reactions can obtain same ranks resulting in different numbers of total ranks in the considered settings. Low rank number corresponds to high FC.(PDF)

Table S5 **Normalized functional centralities for the metabolic function of lactate production under conditions of aerobic respiration (sample size 200,000).**
(PDF)

Table S6 **Normalized functional centralities for the metabolic function of ATP production under conditions of aerobic respiration (sample size 200,000).**
(PDF)

Table S7 **Normalized functional centralities for the metabolic function of lactate production under conditions of nitrate respiration (sample size 200,000).**
(PDF)

Table S8 **Normalized functional centralities for the metabolic function of lactate production under conditions of fermentation (sample size 200,000).**
(PDF)

Table S9 **Normalized functional centralities for the metabolic function of ATP production under conditions of nitrate respiration (sample size 200,000).**
(PDF)

Table S10 **Normalized functional centralities for the metabolic function of ATP production under conditions of fermentation (sample size 200,000).**
(PDF)

Table S11 **Pathways, reaction identifiers, description of reactions and the corresponding gene(s) in the metabolic network model of** ***E. coli's*** **central carbon metabolism.**
(PDF)

Table S12 **Metabolite identifiers and full names of the metabolites in the metabolic network model of** ***E. coli's*** **central carbon metabolism.**
(PDF)

Text S1 **Axiomatization of functional centralities.**
(PDF)

Text S2
**Error calculation for the KO-reduced functionality** 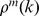 **in the multiple knockout study.**
(PDF)
