# Supplementary material for: Structural Control of Metabolic Flux
Source: PLoS Comput Biol. 2013 Dec 19;9(12):e1003368. doi: 10.1371/journal.pcbi.1003368 (PMC3868538; doi:10.1371/journal.pcbi.1003368)
Supplement: Figure S4 — Functional centralities (FCs) for ATP production under conditions of fermentation. Thickness of arrows corresponds to FCs in the central carbon metabolism of E. coli. (PDF) [file pcbi.1003368.s004.pdf]

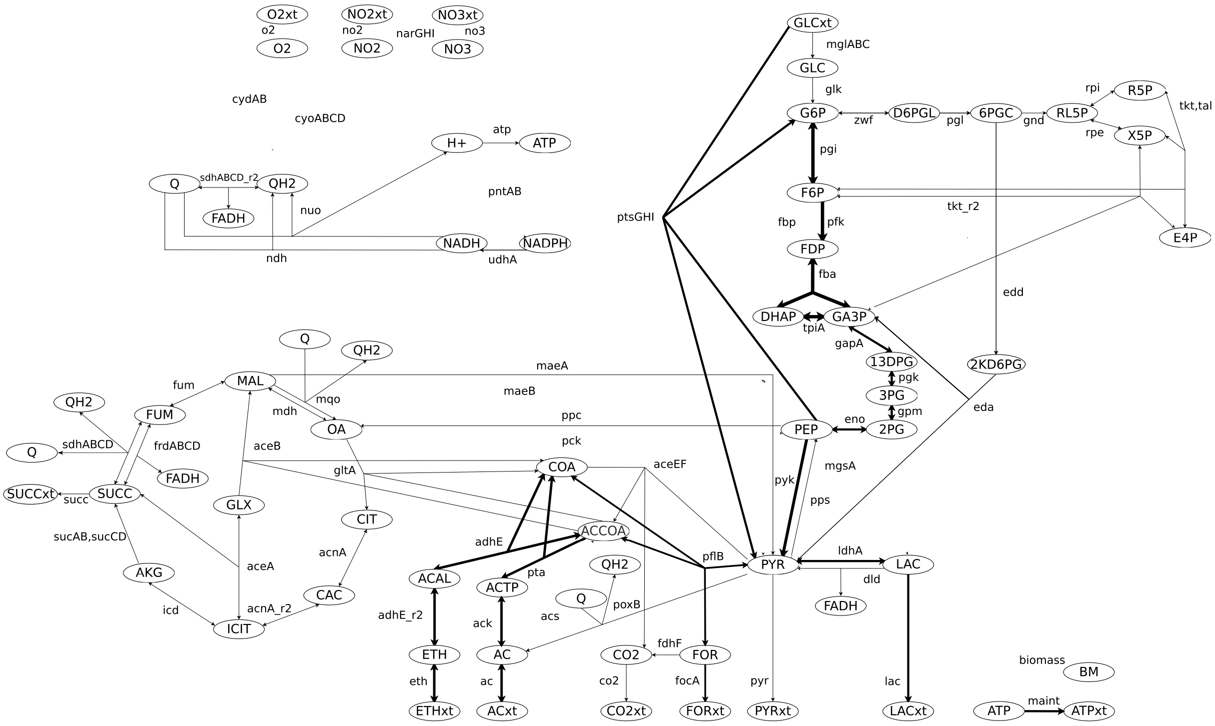

Figure S4: Functional centralities (FCs) for ATP production under conditions of fermentation. Thickness of arrows corresponds to FCs in the central carbon metabolism of *E. coli*.
