# Supplementary material for: Structural Control of Metabolic Flux
Source: PLoS Comput Biol. 2013 Dec 19;9(12):e1003368. doi: 10.1371/journal.pcbi.1003368 (PMC3868538; doi:10.1371/journal.pcbi.1003368)
Supplement: Figure S5 — Elemental coalitions with and without consideration of transporters. Example network containing two EFMs. All considered reactions are shown in black, the blue dashed box marks an elemental coalition. A and B show the case considering transporters; the elemental coalitions equal the reaction sets of the two EFMs and . C and D show the case not considering transporters; the reaction set of the EFMs are reduced to and , which is the only elemental coalition, since . (PDF) [file pcbi.1003368.s005.pdf]

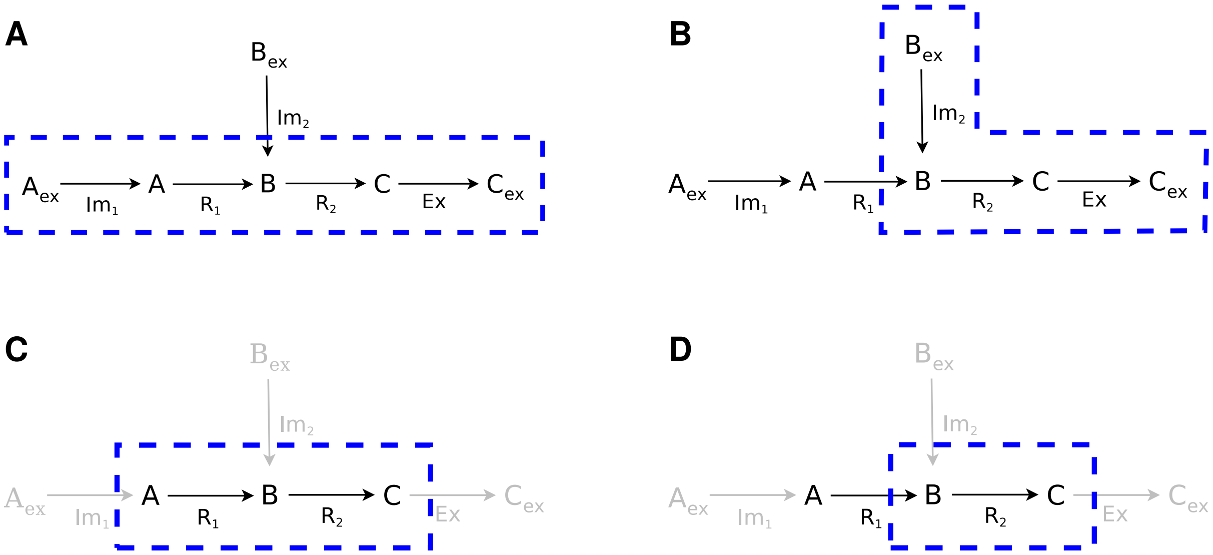

Figure S5: Elemental coalitions with and without consideration of transporters. Example network containing two EFMs. All considered reactions are shown in black, the blue dashed box marks an elemental coalition. **A** and **B** show the case considering transporters; the elemental coalitions equal the reaction sets of the two EFMs $R_{EFM_1} = \{Im_1, R_1, R_2, Ex\} = EC_1$ and $R_{EFM_2} = \{Im_2, R_2, Ex\} = EC_2$. **C** and **D** show the case not considering transporters; the reaction set of the EFMs are reduced to $R'_{EFM_1} = \{R_1, R_2\}$ and $R'_{EFM_2} = \{R_2\} = EC$, which is the only elemental coalition, since $R'_{EFM_2} \subseteq R'_{EFM_1}$.
