# Supplementary material for: Structural Control of Metabolic Flux
Source: PLoS Comput Biol. 2013 Dec 19;9(12):e1003368. doi: 10.1371/journal.pcbi.1003368 (PMC3868538; doi:10.1371/journal.pcbi.1003368)
Supplement: Table S1 — Normalized functional centralities for the metabolic function of biomass production under conditions of aerobic respiration (sample size 200,000). (PDF) [file pcbi.1003368.s006.pdf]

Table S1: Normalized functional centralities for the metabolic function of biomass production under conditions of aerobic respiration (sample size 200,000).

Rank	Reaction ID	FC	Error
1	atp	0.15472830	0.00062984
2	nuo	0.04850746	0.00020154
3	fba	0.03301666	0.00023341
	pfk	0.03266578	0.00023206
	eda	0.03221566	0.00025301
	edd	0.03211489	0.00025231
4	cyoABCD	0.02946609	0.00014196
5	tkt	0.02499892	0.00018511
	tal	0.02497128	0.00018470
6	o2	0.02350761	0.00020785
7	tpiA	0.02230088	0.00016156
8	co2	0.01989963	0.00012298
9	fumA	0.01873015	0.00018359
10	gnd	0.01824617	0.00013532
11	pgl	0.01776700	0.00012986
	zwf	0.01761148	0.00012860
12	gapA	0.01728920	0.00010215
	eno	0.01725905	0.00010311
	pgk	0.01722994	0.00010329
	gpm	0.01718744	0.00010247
13	ac	0.01522833	0.00014525
14	rpe	0.01454131	0.00005381
	tkt_r2	0.01447099	0.00005115
	pgi	0.01439366	0.00004857
15	aceEF	0.01386067	0.00003963
	ppc	0.01381703	0.00002155
	acnA	0.01381444	0.00002102
	acnA_r2	0.01381444	0.00002102
	biomass	0.01381444	0.00002102
	gltA	0.01381444	0.00002102
	icd	0.01381444	0.00002102
	rpiA	0.01381444	0.00002102
16	ptsGHI	0.01370178	0.00002513
17	udhA	0.01304183	0.00007760
18	sucAB	0.01274552	0.00016867
	sucCD	0.01268103	0.00016723
19	pps	0.01149624	0.00004291
20	pntAB	0.01014734	0.00007768
21	ack	0.00957259	0.00011898
	aceA	0.00957086	0.00014885
	pta	0.00952720	0.00011861
	aceB	0.00942610	0.00014783
22	sdhABCD	0.00825935	0.00013359
23	cydAB	0.00782560	0.00009347
	pyk	0.00781245	0.00005939
24	pyr	0.00712105	0.00005069
25	succ	0.00680080	0.00010812
26	ndh	0.00654181	0.00005627
27	glk	0.00544727	0.00004088
	mglABC	0.00541617	0.00004085
28	poxB	0.00486919	0.00006726
29	mdh	0.00401846	0.00006774
30	mgsA	0.00378270	0.00008826
31	sdhABCD_r2	0.00347875	0.00008736
32	frdABCD	0.00315032	0.00008184
33	ldhA	0.00259160	0.00006784
	narGHI	0.00248946	0.00010776
	no2	0.00243732	0.00010569
	no3	0.00242533	0.00010595
34	mqo	0.00219826	0.00006227
35	maeB	0.00189227	0.00005634
36	pck	0.00163297	0.00002380
37	pflB	0.00155995	0.00002461
38	maint	0.00149959	0.00001618
39	fdhF	0.00095790	0.00002079
	dld	0.00095250	0.00005075
	focA	0.00091891	0.00001237
40	maeA	0.00085520	0.00004354
41	adhE	0.00046945	0.00000457
	eth	0.00046924	0.00000455
	adhE_r2	0.00046908	0.00000446
42	acs	0.00044245	0.00000776
43	fbp	0.00025007	0.00001788
44	lac	0.00014180	0.00000422
