# Supplementary material for: Structural Control of Metabolic Flux
Source: PLoS Comput Biol. 2013 Dec 19;9(12):e1003368. doi: 10.1371/journal.pcbi.1003368 (PMC3868538; doi:10.1371/journal.pcbi.1003368)
Supplement: Table S2 — Normalized functional centralities for the metabolic function of biomass production under conditions of nitrate respiration (sample size 200,000). (PDF) [file pcbi.1003368.s007.pdf]

Table S2: Normalized functional centralities for the metabolic function of biomass production under conditions of nitrate respiration (sample size 200,000).

Rank	Reaction ID	FC	Error
1	no2	0.12667865	0.00069077
	narGHI	0.12649428	0.00069072
	no3	0.12530616	0.00068763
2	atp	0.11364859	0.00060060
3	nuo	0.04710564	0.00027668
4	co2	0.02977754	0.00024094
5	pfk	0.02683815	0.00014603
	fba	0.02672737	0.00014588
6	tpiA	0.02604361	0.00013814
7	ac	0.02233669	0.00014736
8	ack	0.02107396	0.00013824
	pta	0.02093709	0.00013738
9	fumA	0.01266424	0.00013517
10	tal	0.01175434	0.00014666
	tkt	0.01175433	0.00014786
11	pyk	0.00982225	0.00007445
12	eda	0.00741893	0.00007583
	edd	0.00739655	0.00007539
13	pntAB	0.00716011	0.00007959
14	gnd	0.00702165	0.00005510
15	pgl	0.00680422	0.00004717
	zwf	0.00676600	0.00004552
	pgi	0.00673621	0.00003162
	sucCD	0.00665018	0.00011616
	rpe	0.00664889	0.00003254
	tkt_r2	0.00664588	0.00003347
	sucAB	0.00653283	0.00011317
	gpm	0.00651658	0.00002196
	gapA	0.00651215	0.00002211
	pgk	0.00650482	0.00002115
	eno	0.00649097	0.00002144
16	acnA	0.00640029	0.00000966
	acnA_r2	0.00640029	0.00000966
	biomass	0.00640029	0.00000966
	gltA	0.00640029	0.00000966
	icd	0.00640029	0.00000966
	ppc	0.00640029	0.00000966
16	ptsGHI	0.00640002	0.00001188
	rpiA	0.00640029	0.00000966
17	udhA	0.00605588	0.00001861
18	pps	0.00580020	0.00001976
19	pflB	0.00521903	0.00001248
20	focA	0.00514543	0.00000731
21	adhE	0.00509473	0.00000495
	adhE_r2	0.00509455	0.00000495
	eth	0.00509455	0.00000495
22	mdh	0.00451369	0.00006373
23	sdhABCD	0.00422367	0.00008789
	succ	0.00422047	0.00006754
	aceA	0.00421170	0.00009558
	pyr	0.00416206	0.00005005
	aceB	0.00413340	0.00009397
24	aceEF	0.00388036	0.00001991
25	ndh	0.00278099	0.00008182
	mglABC	0.00276778	0.00002745
	glk	0.00275491	0.00002684
26	fdhF	0.00260509	0.00001092
27	sdhABCD_r2	0.00198174	0.00006253
28	frdABCD	0.00182574	0.00005968
	mqo	0.00182296	0.00006498
29	maeB	0.00102780	0.00003401
	poxB	0.00097330	0.00003913
	ldhA	0.00089740	0.00004897
30	dld	0.00068245	0.00004606
31	mgsA	0.00028115	0.00002478
	maeA	0.00028090	0.00001953
32	pck	0.00019281	0.00001227
33	maint	0.00011241	0.00000472
34	fbp	0.00008519	0.00001261
35	lac	0.00006802	0.00000212
36	acs	0.00003875	0.00000237
37	cyoABCD	0.00000000	0.00000896
	cydAB	0.00000000	0.00000896
	o2	0.00000000	0.00000896
