# Supplementary material for: Structural Control of Metabolic Flux
Source: PLoS Comput Biol. 2013 Dec 19;9(12):e1003368. doi: 10.1371/journal.pcbi.1003368 (PMC3868538; doi:10.1371/journal.pcbi.1003368)
Supplement: Table S3 — Normalized functional centralities for the metabolic function of biomass production under conditions of fermentation (sample size 200,000). (PDF) [file pcbi.1003368.s008.pdf]

Table S3: Normalized functional centralities for the metabolic function of biomass production under conditions of fermentation (sample size 200,000).

Rank	Reaction ID	FC	Error
1	tpiA	0.08832946	0.00039954
	fba	0.08784966	0.00039853
	pfk	0.08755749	0.00039896
2	ac	0.07647494	0.00033023
	ack	0.07613371	0.00032984
	pta	0.07611889	0.00033011
3	pyk	0.03223412	0.00018902
4	succ	0.01443767	0.00010791
	pgi	0.01431408	0.00004816
5	pflB	0.01344303	0.00000541
	focA	0.01343904	0.00000417
	acnA	0.01343902	0.00000412
	acnA_r2	0.01343902	0.00000412
	adhE_r2	0.01343902	0.00000412
	adhE	0.01343902	0.00000412
	biomass	0.01343902	0.00000412
	eno	0.01343902	0.00000412
	eth	0.01343902	0.00000412
	gapA	0.01343902	0.00000412
	gpm	0.01343902	0.00000412
	gltA	0.01343902	0.00000412
	icd	0.01343902	0.00000412
	pgk	0.01343902	0.00000412
	ppc	0.01343902	0.00000412
	ptsGHI	0.01343902	0.00000412
	rpiA	0.01343902	0.00000412
6	tkt_r2	0.01339796	0.00001040
7	rpe	0.01335614	0.00001120
8	zwf	0.01324055	0.00002903
	pgl	0.01322581	0.00002883
9	gnd	0.01310772	0.00003011
10	udhA	0.01266109	0.00000774
11	eda	0.01235104	0.00001135
	edd	0.01234878	0.00001010
	pps	0.01233223	0.00000911
12	fumA	0.01132433	0.00008937
13	nuo	0.01106120	0.00008761
14	mdh	0.00992722	0.00007682
15	co2	0.00937362	0.00004717
16	pntAB	0.00777133	0.00006925
	tal	0.00768997	0.00006255
17	tkt	0.00756216	0.00006158
18	fdhF	0.00665761	0.00001978
	pyr	0.00664420	0.00002100
	aceEF	0.00661798	0.00001980
19	sucAB	0.00259553	0.00004425
	sucCD	0.00254202	0.00004369
20	sdhABCD	0.00242761	0.00004723
21	atp	0.00188895	0.00005434
22	mglABC	0.00170081	0.00002163
	glk	0.00167745	0.00002148
23	maeB	0.00110652	0.00001735
	frdABCD	0.00107004	0.00003252
	sdhABCD_r2	0.00105955	0.00003174
24	aceA	0.00036028	0.00001642
	aceB	0.00034184	0.00001559
	maeA	0.00032566	0.00000676
25	ldhA	0.00015204	0.00000590
26	lac	0.00014022	0.00000403
27	mqo	0.00001094	0.00000372
	ndh	0.00001084	0.00000371
	dld	0.00000705	0.00000279
	fbp	0.00000644	0.00000091
	poxB	0.00000461	0.00000274
	pck	0.00000159	0.00000053
	maint	0.00000153	0.00000052
28	mgsA	0.00000011	0.00000015
	acs	0.00000000	0.00000713
	cydAB	0.00000000	0.00000713
	cyoABCD	0.00000000	0.00000713
	narGHI	0.00000000	0.00000713
	no2	0.00000000	0.00000713
	no3	0.00000000	0.00000713
	o2	0.00000000	0.00000713
