# Supplementary material for: Structural Control of Metabolic Flux
Source: PLoS Comput Biol. 2013 Dec 19;9(12):e1003368. doi: 10.1371/journal.pcbi.1003368 (PMC3868538; doi:10.1371/journal.pcbi.1003368)
Supplement: Table S4 — Reaction ranks according to functional centrality (FC). The table shows FCs for the metabolic function of lactate production (LAC) under conditions of aerobic respiration (), and ATP production (ATP) under conditions of aerobic respiration (), nitrate respiration () and fermentation (Ferm). Reactions can obtain same ranks resulting in different numbers of total ranks in the considered settings. Low rank number corresponds to high FC. (PDF) [file pcbi.1003368.s009.pdf]

Table S4: Reaction ranks according to functional centrality (FC).

Reaction ID	LAC	ATP		
	O_2	O_2	NO_3	Ferm
glk	7	27	26	14
pgi	2	11	10	3
pfk	2	17	13	2
fba	2	13	11	1
tpiA	4	14	11	2
gapA	1	18	17	7
pgk	1	18	17	7
gpm	1	18	17	7
eno	1	18	17	7
pyk	4	17	14	4
aceEF	7	13	17	12
maeB	7	31	30	15
maeA	7	30	29	15
pck	7	31	30	15
fbp	6	20	21	15
pps	6	29	28	15
zwf	6	17	18	10
pgl	6	17	18	10
gnd	7	11	15	14
rpiA	7	11	15	14
rpe	7	11	15	14
tkt	7	11	15	14
tkt_r2	7	11	15	14
tal	7	11	15	14
gltA	7	6	6	14
acnA	7	6	5	14
acnA_r2	7	6	5	14
icd	7	10	10	15
sucAB	7	10	10	15
sucCD	7	10	10	15
frdABCD	7	21	21	14
fumA	7	7	7	11
mdh	7	12	12	11
mqo	7	23	25	15
aceA	7	11	16	15
aceB	7	11	16	15
ppc	7	29	27	11

Reaction ID	LAC	ATP		
	O_2	O_2	NO_3	Ferm
pntAB	7	26	24	15
udhA	6	9	9	10
atp	7	1	2	11
nuo	7	4	4	11
ndh	7	24	27	15
cyoABCD	7	5	31	15
cydAB	7	17	31	15
narGHI	7	11	1	15
poxB	7	19	23	15
sdhABCD	7	16	19	13
sdhABCD_r2	7	20	21	14
pflB	7	15	16	8
fdhF	7	22	23	14
adhE	7	31	22	5
adhE_r2	7	31	22	5
pta	7	11	11	6
ack	7	11	11	6
acs	7	31	30	15
dld	7	28	29	15
ldhA	1	19	17	7
edd	6	25	22	10
eda	6	25	22	10
mgsA	3	32	31	15
ptsGHI	1	19	17	7
co2	7	2	3	14
o2	7	3	31	15
no3	7	11	1	15
no2	7	11	1	15
eth	7	31	22	5
ac	7	8	8	6
succ	7	26	25	11
lac	1	19	17	7
pyr	7	11	19	15
focA	7	21	20	9
mglABC	7	27	26	14
maint	5	19	17	7
biomass	7	33	31	15

The table shows FCs for the metabolic function of lactate production (LAC) under conditions of aerobic respiration (O_2), and ATP production (ATP) under conditions of aerobic respiration (O_2), nitrate respiration (NO_3) and fermentation (Ferm). Reactions can obtain same ranks resulting in different numbers of total ranks in the considered settings. Low rank number corresponds to high FC.
