# Supplementary material for: Structural Control of Metabolic Flux
Source: PLoS Comput Biol. 2013 Dec 19;9(12):e1003368. doi: 10.1371/journal.pcbi.1003368 (PMC3868538; doi:10.1371/journal.pcbi.1003368)
Supplement: Table S6 — Normalized functional centralities for the metabolic function of ATP production under conditions of aerobic respiration (sample size 200,000). (PDF) [file pcbi.1003368.s011.pdf]

Table S6: Normalized functional centralities for the metabolic function of ATP production under conditions of aerobic respiration (sample size 200,000).

Rank	Reaction ID	FC	Error	Rank	Reaction ID	FC	Error
1	atp	0.14761392	0.00077289	17	pfk	0.00649987	0.00003366
2	co2	0.10961561	0.00065303		pgl	0.00648897	0.00013734
3	o2	0.07388633	0.00046988		pyk	0.00637905	0.00003393
4	nuo	0.06931246	0.00038757	18	eno	0.00508922	0.00003152
5	cyoABCD	0.04415086	0.00027511		gpm	0.00508485	0.00003168
6	gltA	0.04093945	0.00037516		gapA	0.00507850	0.00003071
	acnA	0.04087271	0.00037525		pgk	0.00507068	0.00003022
	acnA_r2	0.04044012	0.00037332	19	ptsGHI	0.00441141	0.00000727
7	fumA	0.03642809	0.00033509		ldhA	0.00440919	0.00000435
8	ac	0.01916326	0.00016867		maint	0.00440897	0.00000427
9	udhA	0.01465352	0.00020102		poxB	0.00436289	0.00007764
10	sucCD	0.01263879	0.00017466		lac	0.00433020	0.00000465
	icd	0.01247200	0.00017291	20	fbp	0.00368344	0.00008031
	sucAB	0.01229357	0.00017125		sdhABCD_r2	0.00359185	0.00011436
11	tal	0.01159214	0.00020045	21	focA	0.00320926	0.00005859
	pta	0.01155961	0.00011717		frdABCD	0.00319408	0.00010877
	ack	0.01148714	0.00011696	22	fdhF	0.00279593	0.00007395
	tkt	0.01129745	0.00019675	23	mqo	0.00185046	0.00007191
	tkt_r2	0.01129203	0.00019737	24	ndh	0.00170100	0.00005439
	rpiA	0.01127889	0.00019691	25	edd	0.00151219	0.00000649
	gnd	0.01125603	0.00019728		eda	0.00151143	0.00000647
	rpe	0.01121718	0.00019649	26	pntAB	0.00141800	0.00002760
	no3	0.01117566	0.00020001		succ	0.00137873	0.00004664
	narGHI	0.01112587	0.00019886	27	mglABC	0.00105819	0.00003108
	aceA	0.01109085	0.00020610		glk	0.00103221	0.00002970
	no2	0.01096777	0.00019718	28	dld	0.00093492	0.00004259
	aceB	0.01085430	0.00020302	29	ppc	0.00081758	0.00005040
	pgi	0.01080847	0.00012541		pps	0.00074594	0.00003797
	pyr	0.01064076	0.00010154	30	maeA	0.00064687	0.00004934
12	mdh	0.00972300	0.00010384	31	eth	0.00041041	0.00001146
13	aceEF	0.00854253	0.00014629		adhE_r2	0.00040934	0.00001154
	fba	0.00838021	0.00006482		adhE	0.00040553	0.00001143
14	tpiA	0.00819987	0.00006317		pck	0.00036314	0.00003916
15	pflB	0.00751697	0.00012473		acs	0.00032938	0.00003539
16	sdhABCD	0.00722799	0.00016300		maeB	0.00032868	0.00003641
17	zwf	0.00661768	0.00013910	32	mgsA	0.00011148	0.00000837
	cydAB	0.00661306	0.00013994	33	biomass	0.00000000	0.00001055
