# Supplementary material for: Structural Control of Metabolic Flux
Source: PLoS Comput Biol. 2013 Dec 19;9(12):e1003368. doi: 10.1371/journal.pcbi.1003368 (PMC3868538; doi:10.1371/journal.pcbi.1003368)
Supplement: Table S8 — Normalized functional centralities for the metabolic function of lactate production under conditions of fermentation (sample size 200,000). (PDF) [file pcbi.1003368.s013.pdf]

Table S8: Normalized functional centralities for the metabolic function of lactate production under conditions of fermentation (sample size 200,000).

Rank	Reaction ID	FC	Error
1	eno	0.09089585	0.00000137
	gapA	0.09089585	0.00000137
	gpm	0.09089585	0.00000137
	lac	0.09089585	0.00000137
	ldhA	0.09089585	0.00000137
	pgk	0.09089585	0.00000137
	ptsGHI	0.09089585	0.00000137
2	fba	0.09087546	0.00000516
	pfk	0.09087546	0.00000516
	pgi	0.09087546	0.00000516
3	mgsA	0.09082318	0.00000885
4	pyk	0.00005228	0.00000645
	tpiA	0.00005228	0.00000645
5	maint	0.00003769	0.00000558
6	eda	0.00002038	0.00000427
	edd	0.00002038	0.00000427
	pgl	0.00002038	0.00000427
	udhA	0.00002038	0.00000427
	zwf	0.00002038	0.00000427
	fbp	0.00001962	0.00000420
	pps	0.00001500	0.00000375
7	pck	0.00000036	0.00000092
	ppc	0.00000036	0.00000092
	ac	0.00000000	0.00000101
	aceA	0.00000000	0.00000101
	aceB	0.00000000	0.00000101
	aceEF	0.00000000	0.00000101
	ack	0.00000000	0.00000101
	acnA	0.00000000	0.00000101
	acnA_r2	0.00000000	0.00000101
	acs	0.00000000	0.00000101
	adhE_r2	0.00000000	0.00000101
	adhE	0.00000000	0.00000101
	atp	0.00000000	0.00000101
	biomass	0.00000000	0.00000101
	co2	0.00000000	0.00000101
	cydAB	0.00000000	0.00000101
7	cyoABCD	0.00000000	0.00000101
	dld	0.00000000	0.00000101
	eth	0.00000000	0.00000101
	fdhF	0.00000000	0.00000101
	focA	0.00000000	0.00000101
	frdABCD	0.00000000	0.00000101
	fumA	0.00000000	0.00000101
	glk	0.00000000	0.00000101
	gltA	0.00000000	0.00000101
	gnd	0.00000000	0.00000101
	icd	0.00000000	0.00000101
	maeA	0.00000000	0.00000101
	maeB	0.00000000	0.00000101
	mdh	0.00000000	0.00000101
	mglABC	0.00000000	0.00000101
	mqo	0.00000000	0.00000101
	narGHI	0.00000000	0.00000101
	ndh	0.00000000	0.00000101
	no2	0.00000000	0.00000101
	no3	0.00000000	0.00000101
	nuo	0.00000000	0.00000101
	o2	0.00000000	0.00000101
	pflB	0.00000000	0.00000101
	pntAB	0.00000000	0.00000101
	poxB	0.00000000	0.00000101
	pta	0.00000000	0.00000101
	pyr	0.00000000	0.00000101
	rpe	0.00000000	0.00000101
	rpiA	0.00000000	0.00000101
	sdhABCD	0.00000000	0.00000101
	sdhABCD_r2	0.00000000	0.00000101
	sucAB	0.00000000	0.00000101
	succ	0.00000000	0.00000101
	sucCD	0.00000000	0.00000101
	tal	0.00000000	0.00000101
	tkt	0.00000000	0.00000101
	tkt_r2	0.00000000	0.00000101
