# Supplementary material for: Structural Control of Metabolic Flux
Source: PLoS Comput Biol. 2013 Dec 19;9(12):e1003368. doi: 10.1371/journal.pcbi.1003368 (PMC3868538; doi:10.1371/journal.pcbi.1003368)
Supplement: Table S9 — Normalized functional centralities for the metabolic function of ATP production under conditions of nitrate respiration (sample size 200,000). (PDF) [file pcbi.1003368.s014.pdf]

Table S9: Normalized functional centralities for the metabolic function of ATP production under conditions of nitrate respiration (sample size 200,000).

Rank	Reaction ID	FC	Error
1	no2	0.11456410	0.00074622
	narGHI	0.11436150	0.00074403
	no3	0.11324071	0.00074176
2	atp	0.10444475	0.00069457
3	co2	0.07680516	0.00056743
4	nuo	0.06483717	0.00045248
5	acnA_r2	0.03059286	0.00031784
	acnA	0.03039733	0.00031570
6	gltA	0.02976628	0.00031188
7	fumA	0.02747804	0.00028270
8	ac	0.01265283	0.00013844
9	udhA	0.01131270	0.00015961
10	sucAB	0.01078649	0.00014839
	pgi	0.01078439	0.00009966
	sucCD	0.01076002	0.00014841
	icd	0.01071401	0.00015010
11	fba	0.00966007	0.00005622
	pta	0.00965014	0.00010930
	tpiA	0.00964318	0.00005653
	ack	0.00960009	0.00010915
12	mdh	0.00900856	0.00009442
13	pfk	0.00861545	0.00004157
14	pyk	0.00811305	0.00003914
15	tkt_r2	0.00719432	0.00015323
	tal	0.00713578	0.00015213
	rpiA	0.00708924	0.00015150
	tkt	0.00706082	0.00015165
	gnd	0.00699634	0.00015007
	rpe	0.00695597	0.00015051
16	pflB	0.00648389	0.00011346
	aceA	0.00645946	0.00015441
	aceB	0.00635558	0.00015289
17	gpm	0.00577300	0.00000720
	eno	0.00577119	0.00000687
	pgk	0.00577038	0.00000676
	gapA	0.00576957	0.00000666
	ptsGHI	0.00576775	0.00000586
17	ldhA	0.00576620	0.00000552
	maint	0.00576620	0.00000552
	lac	0.00576588	0.00000553
	aceEF	0.00563772	0.00012600
18	zwf	0.00495348	0.00010254
	pgl	0.00478079	0.00009916
19	pyr	0.00448842	0.00007056
	sdhABCD	0.00447047	0.00012610
20	focA	0.00348836	0.00005609
21	fbp	0.00221053	0.00005849
	frdABCD	0.00212929	0.00008825
	sdhABCD_r2	0.00210527	0.00008698
22	eda	0.00192538	0.00000558
	edd	0.00192513	0.00000556
	eth	0.00190502	0.00002766
	adhE_r2	0.00190108	0.00002766
	adhE	0.00189639	0.00002762
23	fdhF	0.00175182	0.00006093
	poxB	0.00165049	0.00004867
24	pntAB	0.00104123	0.00002694
25	mqo	0.00094446	0.00005038
	succ	0.00092783	0.00003794
26	glk	0.00070101	0.00002423
	mglABC	0.00068824	0.00002408
27	ndh	0.00059311	0.00003135
	ppc	0.00056638	0.00003896
28	pps	0.00041218	0.00002720
29	dld	0.00034296	0.00002487
	maeA	0.00033359	0.00003531
30	pck	0.00021921	0.00003039
	acs	0.00018344	0.00002738
	maeB	0.00014603	0.00002360
31	mgsA	0.00001022	0.00000389
	biomass	0.00000000	0.00001038
	cydAB	0.00000000	0.00001038
	cyoABCD	0.00000000	0.00001038
	o2	0.00000000	0.00001038
