# Supplementary material for: Structural Control of Metabolic Flux
Source: PLoS Comput Biol. 2013 Dec 19;9(12):e1003368. doi: 10.1371/journal.pcbi.1003368 (PMC3868538; doi:10.1371/journal.pcbi.1003368)
Supplement: Table S10 — Normalized functional centralities for the metabolic function of ATP production under conditions of fermentation (sample size 200,000). (PDF) [file pcbi.1003368.s015.pdf]

Table S10: Normalized functional centralities for the metabolic function of ATP production under conditions of fermentation (sample size 200,000).

Rank	Reaction ID	FC	Error
1	fba	0.06045614	0.00027952
2	pfk	0.05977451	0.00027718
	tpiA	0.05969322	0.00027689
3	pgi	0.05909062	0.00027337
4	pyk	0.05163396	0.00023353
5	eth	0.04416550	0.00032530
	adhE	0.04391074	0.00032448
	adhE_r2	0.04375420	0.00032402
6	ac	0.04306452	0.00031912
	ack	0.04282087	0.00031826
	pta	0.04257252	0.00031749
7	gapA	0.03848502	0.00003683
	gpm	0.03848502	0.00003683
	eno	0.03848502	0.00003683
	maint	0.03848502	0.00003683
	pgk	0.03848502	0.00003683
	ptsGHI	0.03848502	0.00003683
	lac	0.03848471	0.00003683
	ldhA	0.03848471	0.00003683
8	pflB	0.03115499	0.00025414
9	focA	0.03036417	0.00024882
10	eda	0.01279228	0.00003682
	edd	0.01279228	0.00003682
	pgl	0.01279228	0.00003682
	udhA	0.01279228	0.00003682
	zwf	0.01279228	0.00003682
11	succ	0.00217079	0.00006324
	ppc	0.00212926	0.00006167
	fumA	0.00208163	0.00006029
	mdh	0.00205938	0.00005926
	nuo	0.00204289	0.00005654
	atp	0.00196387	0.00005527
12	aceEF	0.00096340	0.00004245
13	sdhABCD	0.00042816	0.00002854
14	frdABCD	0.00017550	0.00001904
	sdhABCD_r2	0.00016882	0.00001755
	rpiA	0.00015165	0.00001672
14	gnd	0.00013407	0.00001580
	tkt_r2	0.00012482	0.00001536
	glk	0.00011870	0.00000976
	tkt	0.00011839	0.00001494
	rpe	0.00011807	0.00001485
	tal	0.00011795	0.00001502
	mglABC	0.00010773	0.00000933
	fdhF	0.00010585	0.00001681
	co2	0.00008662	0.00001250
	acnA	0.00006517	0.00001002
	gltA	0.00005991	0.00000981
	acnA_r2	0.00005979	0.00000873
15	pyr	0.00003256	0.00000876
	sucAB	0.00002959	0.00000683
	sucCD	0.00002685	0.00000589
	icd	0.00002184	0.00000556
	aceA	0.00001594	0.00000693
	aceB	0.00001261	0.00000610
	dld	0.00000472	0.00000370
	maeA	0.00000356	0.00000100
	mqo	0.00000083	0.00000214
	ndh	0.00000083	0.00000214
	poxB	0.00000083	0.00000214
	pps	0.00000048	0.00000122
	acs	0.00000000	0.00000556
	biomass	0.00000000	0.00000556
	cydAB	0.00000000	0.00000556
	cyoABCD	0.00000000	0.00000556
	fbp	0.00000000	0.00000556
	maeB	0.00000000	0.00000556
	mgsA	0.00000000	0.00000556
	narGHI	0.00000000	0.00000556
	no2	0.00000000	0.00000556
	no3	0.00000000	0.00000556
	o2	0.00000000	0.00000556
	pck	0.00000000	0.00000556
	pntAB	0.00000000	0.00000556
