# Supplementary material for: Structural Control of Metabolic Flux
Source: PLoS Comput Biol. 2013 Dec 19;9(12):e1003368. doi: 10.1371/journal.pcbi.1003368 (PMC3868538; doi:10.1371/journal.pcbi.1003368)
Supplement: Table S11 — Pathways, reaction identifiers, description of reactions and the corresponding gene(s) in the metabolic network model of E. coli's central carbon metabolism. (PDF) [file pcbi.1003368.s016.pdf]

Table S11: Pathways, reaction identifiers, description of reactions and the corresponding gene(s) in the metabolic network model of *E. coli's* central carbon metabolism.

Pathway	Reaction ID	Reaction			Gene
Glycolysis	*glk*	GLC + ATP	⟶	G6P	*glk*
	pgi	G6P	⟷	F6P	*pgi*
	pfk	ATP + F6P	⟶	FDP	*pfkA/pfkB*
	fba	FDP	⟷	DHAP + GA3P	*fbaA/fbaB*
	tpiA	DHAP	⟷	GA3P	*tpiA*
	gapA	GA3P	⟷	NADH + 13DPG	*gapA*
	pgk	13DPG	⟷	ATP + 3PG	*pgk*
	gpm	3PG	⟷	2PG	*gpmA/gpmB*
	eno	2PG	⟷	PEP	*eno*
	pyk	PEP	⟶	ATP + PYR	*pykA/pykF*
	aceEF	PYR + COA	⟶	NADH + ACCOA + CO2	*aceEF*
Gluconeo-genesis	*maeB*	MAL	⟶	PYR + CO2 + NADPH	*maeB*
	maeA	MAL	⟶	NADH + PYR + CO2	*maeA*
	pck	ATP + OA	⟶	PEP + CO2	*pck*
	fbp	FDP	⟶	F6P	*fbp/glpX*
	pps	2 ATP + PYR	⟶	PEP	*pps*
Pentose phosphate pathway	*zwf*	G6P	⟷	NADPH + D6PGL	*zwf*
	pgl	D6PGL	⟶	6PGC	*pgl*
	gnd	6PGC	⟶	CO2 + NADPH + RL5P	*gnd*
	rpi	RL5P	⟷	R5P	*rpiA/rpiB*
	rpe	RL5P	⟷	X5P	*rpe*
	tkt	R5P + X5P	⟷	GA3P + S7P	*tktA/tktB*
	tkt_r2	X5P + E4P	⟷	F6P + GA3P	*tktA/tktB*
	tal	GA3P + S7P	⟷	F6P + E4P	*talA/talA*
Tricarboxylic acid cycle, glyoxylate cycle	*gltA*	ACCOA + OA	⟶	COA + CIT	*gltA/prpC*
	acnA	CIT	⟷	CAC	*acnA/acnB*
	acnA_r2	CAC	⟷	ICIT	*acnA/acnB*
	icd	ICIT	⟷	CO2 + NADPH + AKG	*icd*
	sucAB	COA + AKG	⟶	NADH + CO2 + SUCCOA	*sucAB*
	sucCD	SUCCOA	⟷	ATP + COA + SUCC	*sucCD*
	frdABCD	SUCC	⟷	FUM + FADH	*frdABCD/sdhAB*
	fum	FUM	⟷	MAL	*fumA/fumB/fumC*
	mdh	MAL	⟷	NADH + OA	*mdh*
	mqo	MAL + Q	⟶	OA + QH2	*mqo*
	aceA	ICIT	⟷	SUCC + GLX	*aceA*
	aceB	ACCOA + GLX	⟶	COA + MAL	*aceB*
A	*ppc*	PEP + CO2	⟶	OA	*ppc*
Respiration	*pntAB*	NADH + Hxt	⟶	NADPH	*pntAB*
	udhA	NADPH	⟶	NADH	*udhA*
	atp	4 Hxt	⟶	ATP	*atpA-I*
	nuo	NADH + Q	⟶	QH2 + 4 Hxt	*nuoA-N*
	ndh	NADH + Q	⟶	QH2	*ndh*
	cyoABCD	QH2 + 0.5 O2	⟶	Q + 4 Hxt	*cyoABCD*
	cydAB	QH2 + 0.5 O2	⟶	Q + 2 Hxt	*cydAB*
	narGHI	QH2 + NO3	⟶	Q + 2 Hxt + NO2	*narGHI*
	poxB	PYR + Q	⟶	CO2 + QH2 + AC	*poxB*
	sdhABCD	SUCC + Q	⟷	FUM + QH2	*sdhABCD*
	sdhABCD_r2	FADH + Q	⟷	QH2	*sdhABCD_r2*
Fermentation	*pflB*	PYR + COA	⟷	ACCOA + FOR	*pflB/tdcE*
	fdhF	FOR	⟶	NADH + CO2	*fdhF/fdoGHI/fdnGHI*
	adhE	NADH + ACCOA	⟷	COA + ACAL	*adhE/mhpF*
	adhE_r2	NADH + ACAL	⟷	ETH	*adhE/adhP/adhC*
	pta	ACCOA	⟶	COA + ACTP	*pta*
	ack	ACTP	⟷	ATP + AC	*ackA/ackB/tdcD/purT*
	acs	2 ATP + COA + AC	⟶	ACCOA	*acs*
	dld	LAC	⟶	PYR + FADH	*dld*
	ldhA	LAC	⟷	NADH + PYR	*ldhA*
EDP	*edd*	6PGC	⟶	2KD6PG	*edd*
	eda	2KD6PG	⟶	GA3P + PYR	*eda*
MP	*mgsA*	DHAP	⟶	LAC	*mgsA*
Transporters	*ptsGHI*	PEP + GLCxt	⟶	G6P + PYR	*ptsGHI*
	co2	CO2	⟶	CO2xt	*co2*
	o2	O2xt	⟶	O2	*o2*
	no3	NO3xt	⟶	NO3	*no3*
	no2	NO2	⟶	NO2xt	*no2*
	eth	ETH	⟷	ETHxt	*eth*
	ac	AC	⟷	Acxt	*ac*
	succ	SUCC	⟶	SUCCxt	*succ*
	lac	LAC	⟶	LACxt	*lac*
	pyr	PYR	⟶	PYRxt	*pyr*
	focA	FOR	⟶	FORxt	*focA*
	mglABC	Hxt + GLCxt	⟶	GLC	*mglABC*
	maint	ATP	⟶	ATPxt	*maint*
BMR	*biomass*	40.2 ATP + 0.33 G6P + 0.07 F6P + 0.12 GA3P + 0.86 3PG + 0.77 PEP + 2.94 PYR + 2.41 ACCOA + 15.7 NADPH + 1.65 OA + 0.96 R5P + 0.36 E4P + 1.28 AKG	⟶	3 NADH + 2.41 COA + Biomass	

A – Anaplerotic reaction, EDP – Entner-Doudoroff pathway, MP – Methylgloxal pathway, BMR – Biomass reaction
