# Supplementary material for: Structural Control of Metabolic Flux
Source: PLoS Comput Biol. 2013 Dec 19;9(12):e1003368. doi: 10.1371/journal.pcbi.1003368 (PMC3868538; doi:10.1371/journal.pcbi.1003368)
Supplement: Table S12 — Metabolite identifiers and full names of the metabolites in the metabolic network model of E. coli's central carbon metabolism. (PDF) [file pcbi.1003368.s017.pdf]

Table S12: Metabolite identifiers and full names of the metabolites in the metabolic network model of *E. coli's* central carbon metabolism.

Metabolite ID	Metabolite	Metabolite ID	Metabolite
13DPG	1,3-Diphosphoglycerate	GA3P	Glyceraldehyde 3-phosphate
2KD6PG	2-Keto-3-deoxy-6-phosphogluconate	GLC	Glucose
2PG	2-Phosphoglycerate	GLCxt	Glucose (external)
3PG	3-Phosphoglycerate	GLX	Glyoxylate
6PGC	6-Phosphogluconate	Hxt	Proton (external)
AC	Acetate	ICIT	Isocitrate
ACAL	Acetaldehyde	LAC	Lactate
ACCOA	Acetyl-CoA	LACxt	Lactate (external)
ACTP	Acetylphosphate	MAL	Malate
Acxt	Acetate (external)	NADH	Nicotinamide adenine dinucleotide
AKG	alpha-Ketoglutarate	NADPH	Nicotinamide adenine dinucleotide phosphate
ATP	Adenosine triphosphate	NO2	Nitrite
ATPxt	Adenosine triphosphate (external)	NO2xt	Nitrite (external)
BM	Biomass (external)	NO3	Nitrate
CAC	cis-Aconitate	NO3xt	Nitrate (external)
CIT	Citrate	O2	Oxygen
CO2	Carbon dioxide	O2xt	Oxygen (external)
CO2xt	Carbon dioxide (external)	OA	Oxaloacetate
COA	Coenzyme A	PEP	Phosphoenolpyruvate
D6PGL	6-Phosphogluconolactone	PYR	Pyruvate
DHAP	Dihydroxyacetone phosphate	PYRxt	Pyruvate (external)
E4P	Erythrose 4-phosphate	Q	Ubiquinone
ETH	Ethanol	QH2	Ubiquinol
ETHxt	Ethanol (external)	R5P	Ribose 5-phosphate
F6P	Fructose 6-phosphate	RL5P	Ribulose 5-phosphate
FADH	Flavin adenine dinucleotide	S7P	Sedoheptulose 7-phosphate
FDP	Fructose diphosphate	SUCC	Succinate
FOR	Formate	SUCCOA	Succinyl-CoA
FORxt	Formate (external)	SUCCxt	Succinate (external)
FUM	Fumarate	X5P	Xylose 5-phosphate
G6P	Glucose 6-phosphate		
