# Supplementary material for: Structural Control of Metabolic Flux
Source: PLoS Comput Biol. 2013 Dec 19;9(12):e1003368. doi: 10.1371/journal.pcbi.1003368 (PMC3868538; doi:10.1371/journal.pcbi.1003368)
Supplement: Text S1 — Axiomatization of functional centralities. (PDF) [file pcbi.1003368.s018.pdf]

Structural Control of Metabolic Flux - Supplementary Text S1

Max Sajitz-Hermstein[1,2,*] and Zoran Nikoloski[1]

[1]Systems Biology and Mathematical Modeling Group, Max Planck Institute of Molecular Plant Physiology, 14476 Potsdam, Germany
[2]System Regulation Group, Max Planck Institute of Molecular Plant Physiology, 14476 Potsdam, Germany
[*]E-mail: sajitz@mpimp-golm.mpg.de

Axiomatization of functional centralities

In the following we give axiomatic requirements for functional centrality, such that it provides a fair assignment of the reactions' contributions to a metabolic function of interest. These requirements define a mapping of the corresponding cooperative game $(\mathcal{N}, v)$ with transferable utility and restricted to a family of coalitions $\mathcal{S}$ on the Shapley value for arbitrary families of coalitions [1].

Here a *value* denotes a mapping that assigns to a characteristic function $v \in \mathcal{V}$ and $i \in \mathcal{N}$ a real number $\phi_i(v)$, whereby $\mathcal{V}$ denotes the set of characteristic functions of a family of coalitions $\mathcal{S}$. In our case, the family of coalitions $\mathcal{S}$ corresponds to the set of functional subnetworks with respect to a metabolic function and environmental conditions. The characteristic function v assigns every $S \in \mathcal{S}$ the synthesizing capacity of the corresponding subnetwork with respect to the metabolic function. Remember that the graph $G_{\mathcal{S}} = (\mathcal{S}, \mathcal{A})$ consists of the elements $S \in \mathcal{S}$ as nodes. The set of arcs $\mathcal{A}$ comprises all (S, S') with $S, S' \in \mathcal{S}$ and $S \subsetneq S'$ for which it holds that there exists no $S'' \in \mathcal{S}$ with $S \subsetneq S'' \subsetneq S'$. The set $\mathcal{A}_i \subset \mathcal{A}$ consists of all (S, S') with $i \in S' \setminus S$.

Efficiency Functional centrality ϕ is required to be precisely a distribution of the synthesizing capacity of a metabolic function $v(\mathcal{N})$ among the set of considered reactions $\mathcal{N}$. Then functional centrality ϕ_i of a reaction $i \in \mathcal{N}$ denotes a share of the synthesizing capacity. A value ϕ is called *efficient*, if

$$\sum_{i \in \mathcal{N}} \phi_i(v) = v(\mathcal{N}) \text{ for all } v \in \mathcal{V}.$$

Marginalism Functional centrality is required to incorporate all possible interactions of a reaction with the remaining metabolic system. These interactions encompass the effects of activating or deactivating a reaction on the synthesizing capacity of a metabolic network's accessible configurations. Accordingly, the accessible configurations of the metabolic network are determined by unique assignment of active/inactive status to its reactions, which correspond to functional subnetworks. Therefore, functional centrality is required to integrate a reaction's contribution to all functional subnetworks (and to the empty set). A value ϕ is called *marginalist*, if

$$\forall i \in \mathcal{N}, \quad \forall (S, S') \in \mathcal{A}_i : \quad \exists \lambda_i(S, S') \in \mathbb{R}, \text{s.t.}$$
$$\phi_i(v) = \sum_{(S,S') \in \mathcal{A}_i} \lambda_i(S, S')(v(S') - v(S)) \text{ for all } v \in \mathcal{V}.$$

Internal symmetry Functional centrality is required to distribute the contribution of multiple reactions equally among them, meaning that in this case the assignment is irrespective of the reactions' identifiers. A value ϕ is said to have *internal symmetry*, if

$$\lambda_i(S,S') = \lambda_j(S,S') \text{ for all } (S,S') \in \mathcal{A} \text{ and } i,j \in S' \setminus S.$$

In [1] it is shown, that a value for an arbitrary family of coalitions that is *efficient*, *marginalist* and satisfies *internal symmetry*, has the form as described in the Methods Section.

The described value is not unique, a weighting of the maximal chains (paths on $G_{\mathcal{S}}$ from $\emptyset$ to $\mathcal{N}$) can be chosen. In this study we utilize equal weighting. Alternative weightings (by chain length and harmonic weighting) were tested and have shown quantitatively similar results (data not shown).

References

[1] Aguilera NE, Di Marco SC, Escalante MS (2010) The Shapley value for arbitrary families of coalitions. Eur J Oper Res 204: 125-138.
