# Supplementary material for: Structural Control of Metabolic Flux
Source: PLoS Comput Biol. 2013 Dec 19;9(12):e1003368. doi: 10.1371/journal.pcbi.1003368 (PMC3868538; doi:10.1371/journal.pcbi.1003368)
Supplement: Text S2 — Error calculation for the KO-reduced functionality in the multiple knockout study. (PDF) [file pcbi.1003368.s019.pdf]

Structural Control of Metabolic Flux - Supplementary Text S2

Max Sajitz-Hermstein[1,2,*] and Zoran Nikoloski[1]

[1]Systems Biology and Mathematical Modeling Group, Max Planck Institute of Molecular Plant Physiology, 14476 Potsdam, Germany
[2]System Regulation Group, Max Planck Institute of Molecular Plant Physiology, 14476 Potsdam, Germany
*E-mail: sajitz@mpimp-golm.mpg.de

Error calculation for KO-reduced functionality $\rho^{\mathbf{m}}(\mathbf{k})$ in the multiple knockout study

The error calculation for the KO-reduced functionality $\rho^m(k)$ is independent of the measure m. Therefore, we do not consider a specific measure m in the following. The KO-reduced functionality $\rho(k)$ for knockout of k reactions can be written as

$$\rho(k) = \frac{\sum_{i=1}^{R} B_i^k}{\sum_{i=1}^{R} \xi(B_i^k)} =: \frac{P}{V},$$

with

$$\xi(x) = \begin{cases} 1 & \text{if } x > 0 \\ 0 & \text{else.} \end{cases}$$

The relative synthesizing capacity of the metabolic function after k knockouts in the sample i is denoted by B_i^k. In the case of biomass production, the relative synthesizing capacity means the outcome of optimal biomass production after k knockouts divided by the optimal outcome for zero knockouts. Whether the resulting subnetwork is functional or not is indicated by $\xi(B_i^k)$. The denominator ensures that only functional subnetworks are considered. The sample size is denoted by R. The knockouts are determined by sampling without replacement utilizing independent and identically distributed uniform random numbers. Therefore, the B_i^k as well as the $\xi(B_i^k)$ are also independent and identically distributed. The exact value of $\rho(k)$ is the expectation value obtained in the limit $R \to \infty$. In practice, R is finite such that P, V and $\rho(k)$ represent sample means. In the following, we denote the variables for finite R by $\overline{P}$, $\overline{V}$ and $\overline{\rho(k)}$ to distinguish them from the expectation values. Accordingly, we denote the sample means $\overline{B_i^k}$ and $\overline{\xi(B_i^k)}$. The corrected estimates of the variance of $\overline{P} = R \cdot \overline{B_i^k}$ and $\overline{V} = R \cdot \overline{\xi(B_i^k)}$ are [1],

$$\sigma_{\overline{P}}^2 = (R \cdot \sigma_{\overline{B_i^k}})^2 = R^2 \cdot \frac{\sum_{i=1}^{R} \left(B_i^k - \overline{B_i^k} \right)^2}{R(R-1)} \quad \text{and}$$

$$\sigma_{\overline{V}}^2 = (R \cdot \sigma_{\overline{\xi(B_i^k)}})^2 = R^2 \cdot \frac{\sum_{i=1}^{R} \left(\xi(B_i^k) - \overline{\xi(B_i^k)} \right)^2}{R(R-1)}.$$

Error propagation [2] yields an approximation of the variance of KO-reduced functionality $\overline{\rho}$ in the case of finite R,

$$\sigma^2_{\overline{\rho}} \approx \left(\frac{\partial \rho}{\partial P}\bigg|_{P=\overline{P},V=\overline{V}} \right)^2 \sigma^2_{\overline{P}} + \left(\frac{\partial \rho}{\partial V}\bigg|_{P=\overline{P},V=\overline{V}} \right)^2 \sigma^2_{\overline{V}} + 2 \underbrace{\frac{\partial \rho}{\partial P}\bigg|_{P=\overline{P},V=\overline{V}} \cdot \frac{\partial \rho}{\partial V}\bigg|_{P=\overline{P},V=\overline{V}}}_{\left(\frac{1}{\overline{V}}\right)\cdot\left(-\frac{\overline{P}}{\overline{V}^2}\right)} \cdot cov(P,V)$$

$$= \left(\frac{1}{\overline{V}}\right)^2 \sigma^2_{\overline{P}} + \left(\frac{\overline{P}}{\overline{V}^2}\right)^2 \sigma^2_{\overline{V}} - 2\frac{\overline{P}}{\overline{V}^3} \cdot cov(P,V).$$

The covariance is calculated by

$$\begin{aligned} cov(P,V) &= cov\left(\sum_{i=1}^{R} B_i^k, \sum_{i=1}^{R} \xi(B_i^k)\right) \\ &= \sum_{i=1}^{R}\sum_{j=1}^{R} cov\left(B_i^k, \xi(B_j^k)\right) \\ &= \sum_{i=1}^{R} cov\left(B_i^k, \xi(B_i^k)\right) \\ &= R \cdot cov\left(B_i^k, \xi(B_i^k)\right), \end{aligned}$$

whereby we have exploited the properties of identical and independent distribution of B_i^k and $\xi(B_i^k)$. We utilize the error interval $\left[\overline{\rho(k)} - 2\sigma_{\overline{\rho(k)}}, \overline{\rho(k)} + 2\sigma_{\overline{\rho(k)}}\right]$ which comprises the exact value of $\rho(k)$ with a probability larger than 95%.

References

[1] Bronstein I, Semendjajew K, Musiol G, Mühlig H (2001) Taschenbuch der Mathematik. Harri Deutsch Verlag.

[2] Bevington PR, Robinson KD (2002) Data reduction and error analysis for the physical sciences. Mcgraw-Hill Higher Education.
